# Effect of transcriptional delay on ribosome abundance control

**DOI:** 10.1007/s00285-026-02420-3

**Published:** 2026-06-12

**Authors:** Pavan C. Perera, Sergei S. Pilyugin, Lisa Davis, Tomas Gedeon

**Affiliations:** 1 Department of Mathematical Sciences, Montana State University, PO Box 172400, Bozeman, 59717-2400 MT USA; 2 Department of Mathematics, University of Florida, 1400 Stadium Road, Gainesville, 32611-8105 FL USA

**Keywords:** DDE-BIFTOOL, Delay differential equations, Hopf bifurcation, Ribosome abundance, 34K18, 37G15, 37N25, 92C37, 92C42

## Abstract

Time delays are inherent in biological processes such as transcription and translation, and they play a vital role in cellular control by altering timing of feedback loops and therefore stability of steady states. Standard ordinary differential equation (ODE) models neglect these delays, potentially eliminating important dynamic behavior. We present a mathematical model that explores the synthesis of ribosomal proteins and their mRNA in *E. coli* using both ODE and delay differential equation (DDE) models. Comparing these two models we discover that, while there can only be two equilibrium states in both the ODE and the DDE systems, the addition of delays destabilizes the internal equilibrium via the Hopf bifurcation, resulting in oscillatory dynamics. We show that there are parameter combinations where two stable periodic orbits coexist, and one of them terminates in a torus bifurcation giving rise to a stable torus of recurrent orbits. The existence of oscillatory behavior is consistent with experiments.

## Introduction

Delays are ubiquitous in many physical and biological systems. In cellular dynamics, the delays induced by transcription and translation of DNA and mRNA occur every time a new enzyme is needed. This may be unimportant in multicellular organisms where each cell is in a relatively stable environment, but seems vitally important in unicellular organisms (i.e. *E. coli*) where responding rapidly to changes in the environment is crucial for survival.

One of the key determinants of evolutionary success in single cell organisms is their growth rate. Metabolic adaptations in nutrient-rich environments favor maximal growth rate, while in limited resources the growth has to be tailored more carefully. The key ingredient correlated with the growth rate in various organisms is the abundance of ribosomes (Scott et al. 2010, 2014). Ribosomes build proteins, but since ribosomes themselves contain many proteins, they are the root of the self-replicatory nature of life (Reuveni et al. 2017). Since ribosomes are large and therefore expensive complexes to build, the control of their abundance is crucial for evolutionary success of single-cell organisms, particularly those that live in changing environments.

In an earlier paper (Shea et al. 2023) we developed an ODE model for ribosome abundance control in *E. coli*. This model included many known control mechanisms and the subsequent analysis revealed that the corresponding six-dimensional ODE system can operate in two different regimes. Either there is a single stable equilibrium *Q* on the boundary of the positive orthant $R^{6+}$ where there are no free ribosomes, or, in addition to *Q*, there is a single stable equilibrium *P* in the interior of the positive orthant. When *P* exists, *Q* is unstable; the transition between the two regimes happens through a transcritical bifurcation. The paper (Shea et al. 2023) associated the first regime to cellular behavior in limited resources, while the second one to behavior when resources are abundant.

**Fig. 1:**
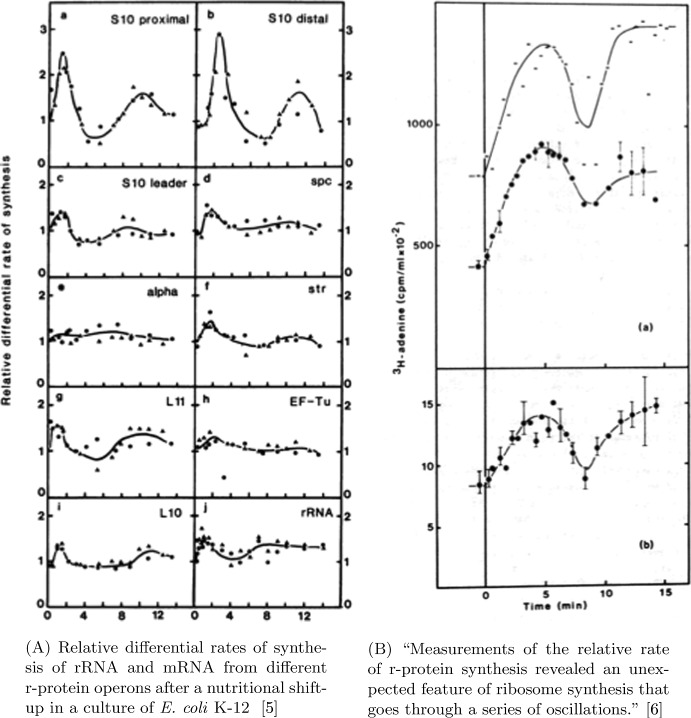
Transcriptional regulation and oscillatory dynamics in ribosomal synthesis

In this paper we continue our investigation of ribosome control in *E. coli* by asking whether delays can destabilize the equilibrium *P*. There is experimental data (see Figure 1) suggesting that under a sudden down shift in resource quality, oscillations are possible. It is well known from control theory that a sufficiently long delay in the negative feedback loop may lead to oscillations. In most engineering applications, these oscillations present an unwanted side effect of negative control. The data in Figure 1 show rapidly decaying oscillations. Since the experiments were done in a culture rather than in single cells, this is a collective measurement of a population of cells. The decaying oscillations on the population level could be caused by de-synchronization of individual cells each of which exhibits sustained oscillation, or it may be that each cell individually exhibits decaying oscillations. The data cannot distinguish between these scenarios. Making a distinction between sustained vs. decaying oscillation is more a mathematical rather than a biological question. Nevertheless, motivated by the question of whether transcriptional and translational delays within the ribosomal abundance control mechanism can cause oscillations, we derive a delay differential equation (DDE) model for ribosome abundance by simultaneously simplifying the six-dimensional ODE model to a three-dimensional ODE model and incorporating delays. The resulting three-dimensional DDE model also accounts for the effect of dilution of molecules in steady growth caused by the increasing volume of the cell, but does not incorporate other control mechanisms such as ribosome deactivation and hibernation (Prossliner et al. 2018).

Our initial analysis of the simplified three-dimensional ODE model shows that it mimics dynamics of the six-dimensional model. Again, there are two regimes, one represented by an equilibrium *Q* on the boundary of $R^{3+}$ and the other “abundant" regime where both *P* and *Q* are present with *P* being an asymptotically stable equilibrium in the interior of $R^{3+}$ and *Q* being unstable.

Analysis of the DDE system recovers these two regimes. However, we make an important observation that the existence of the equilibrium *P* in the abundant regime depends on the value of the delay τ. In particular, for any set of model parameters which supports the abundant regime, the equilibrium *P* only exists for a limited set of delay values $\tau \in [0, \tau _{crit}]$. For each fixed set of model parameters, there is a value $\tau _{crit}$ where the equilibria *P* and *Q* undergo transcritical bifurcation, and for larger values of the delay, that is, $\tau > \tau _{crit}$, the point *Q* is the only non-negative equilibrium. From the biological perspective, this corresponds to the situation when the delay is so long that by the time the mRNA or protein is produced, the volume of the cell grew so much that the resulting diluted concentrations of mRNA and protein are insufficient to sustain the interior state *P*. From the mathematical point of view this makes the challenge of finding oscillations induced by negative feedback significantly harder. It is known that sufficiently large delayed negative feedback can destabilize the system and induce oscillations. However, since the stable equilibrium *P* only exists in our model for a finite range of delays τ, the delay cannot be arbitrary large.

To investigate the possibility of delay-induced instability, we first identify the experimentally supported values of the parameters β, ξ, and μ in *E. coli* (see Table 1), and we place these values in the vector $\zeta \in \mathbb {R}^3$. For the five parameters $\alpha , K, B, \kappa ,$ and *G*, where the values are not available, we estimate their ranges based on experimental data (see Table 1). In order to sample this space, we select five uniformly spaced values from each parameter range, resulting in $5^5 = 3125$ unique parameter combinations, which we call $\mathcal {R}$. For each combination $u \in \mathcal {R}$, we then find the critical delay value, $\tau _{crit}$. Finally, we select 30 uniformly spaced values of τ within the range $J(u):=[0.05\tau _{crit}, 0.95\tau _{crit}]$. Thus, for each of the 3125 parameter combinations $u \in \mathcal {R}$, we associate 30 distinct values of $\tau \in J(u)$, which we call *T*(*u*). Let $T:=\cup _{u\in \mathcal {R}} T(u)$ be the collection of all the τ values. Therefore, our parameter space has the form

$$\begin{aligned} (u,T(u);\zeta ) \in \mathcal {R}\times T\times \zeta , \end{aligned}$$

which has 3125×30=93,750 parameter combinations. For each of these combinations, we compute the eigenvalues of the linearization at *P* and identify those parameters where the leading eigenvalue pair is a complex conjugate pair with positive real part. It suffices to locate a parameter combination for which *P* is simply unstable. We have proved that *P* becomes stable when the delay τ either decreases to zero or increases to $\tau _{crit}$. We also proved that *P* cannot undergo a steady-state bifurcation because the zero eigenvalue does not occur. Combining these properties, we are essentially guaranteed that continuing an initially unstable *P* in either direction of τ will produce a Hopf point.

Starting from such a parameter combination, we use the MATLAB package DDE Biftool (Sieber et al. 2017) to execute a one-parameter continuation along τ in both directions until we find the nearest *Hopf point*. Note that we will use the term *Hopf point* to denote a parameter value at which a linearization of *P* has purely imaginary eigenvalues $\pm \beta i$ with $\beta \not = 0$, and it will be Hopf points that we will discuss in our analysis. When we use the software DDE Biftool to numerically find these points, the software also verifies the non-degeneracy conditions that imply that the pair of eigenvalues is crossing at non-zero speed, and that the Lyapunov coefficient is non-zero. Only if such a crossing results in birth of a non-degenerate periodic orbit and will be called a *Hopf bifurcation.*

With our initial sampling of the parameter space, the Biftool computations indicate that there are no Hopf points in the parameter space $\mathcal {R}\times T\times \zeta$. However, if we increase the value of the parameter ξ (decay rate of the ribosomal proteins) one hundred fold, forming a new set of values $\bar{\zeta }$, then we find that for approximately 0.07% of points $u \in \mathcal {R}\times T\times \bar{\zeta }$, there exists a nearby Hopf bifurcation. In fact, for such *u* there is always a pair of Hopf bifurcations at $\tau _1 < \tau _2$. For these pairs, we observe that the direction of crossing of the imaginary axis is different at the two values: at $\tau _1$ a complex pair of eigenvalues moves from the negative to the positive half-plane. At $\tau _2$ the eigenvalues cross the imaginary axis moving from the positive to the negative half-plane as τ increases. Therefore, at $\tau _1$ the equilibrium *P* generically undergoes a Hopf bifurcation and becomes unstable, while at $\tau _2$ it regains stability. Numerically, we find that both Hopf bifurcations are supercritical and thus a stable periodic orbit exists for $\tau \in [\tau _1, \tau _2]$. From the periodic orbit continuation, we observe that there is a continuous branch of periodic orbits that terminates at $\tau _1$ and $\tau _2$. We call such a pair of Hopf bifurcations $[\tau _1, \tau _2]$ connected by a branch of periodic orbits a *Hopf Pair*.

To supplement the relatively low density of sampling of the parameter space $\mathcal {R}$, we perform a series of two-parameter continuations of Hopf bifurcations in various pairings of parameters $(\alpha , K, B, \kappa , G)\times \bar{\zeta }$. We confirm that the Hopf bifurcations are connected by curves of Hopf bifurcations in the two-dimensional sections of the five-dimensional parameter space. Combining these two-parameter continuations we observe that the Hopf bifurcations occur along approximately two-dimensional manifolds in the five-dimensional space of parameters $(\alpha , K, B, \kappa , G)\times \bar{\zeta }$.

We investigate the possible existence of more than two Hopf bifurcations at the same parameter values in $\mathcal {R}$, but at different values of τ, by first sampling at a large set of values within the parameter set $\bar{\zeta }$. After extensive analytical reformulation of the problem of finding parameters where the linearization at *P* has purely imaginary eigenvalues, we numerically show that when we allow the maximal growth rate μ to vary between $\mu = 7 \times 10^{-5}$ and $\mu = 5 \times 10^{-5}$, there are multiple Hopf bifurcations where more than a single pair of imaginary eigenvalues cross the imaginary axis, with each subsequent bifurcation changing the dimension of the unstable manifold of the equilibrium *P* in increments/decrements of 2. In particular, we show that there are parameter combinations for which there exists a non-empty interval of τ values where there are two co-existing stable periodic orbits, and that this interval terminates in a torus bifurcation where a stable torus emerges from one of the periodic orbits.

Motivated by the difficulty to distinguish true oscillations and decaying oscillations in experimental time series of finite length, in the second set of computations we also investigate the prevalence of decaying oscillations in the parameter space. We count the number of points in parameter space $(u,\tau ; \zeta ) \in \mathcal {R}\times T\times \zeta$ as well as $(u,\tau ; \zeta ) \in \mathcal {R}\times T \times \bar{\zeta }$ where there is at least one value of $\tau \in T(u)$ for which the leading eigenvalue (i.e one with the largest real part) of the linearization at *P* is a complex pair. Surprisingly, about 70% of parameters $\in \mathcal {R} \times T\times \bar{\zeta }$ but also over 50% of parameters $\in \mathcal {R}\times T\times \zeta$ have leading pair of complex eigenvalues. Therefore, while Hopf bifurcations are rare, the decaying oscillations are highly prevalent in this system.

To assess whether the (decaying) oscillations generated by the transcriptional and translation delay in the ribosome abundance feedback loop are biologically relevant, we note several important features of the predicted oscillations. First, as mentioned above, within the range of estimated biological parameters the equilibrium *P* is always stable. Second, only when the ribosomal protein decay rate is increased so that the protein half-life is about 0.5 minutes, which is consistent with the rate for most unstable *E. coli* proteins (Nagar et al. 2021), we find sustained oscillations. However, it is known that the ribosomal proteins are very stable (Piir et al. 2011). Third, the cellular growth rate induced dilution of all concentrations imposes an upper limit $\tau _{crit}$ beyond which the equilibrium *P* is always stable. Finally, for all parameter sets that we tested, whether the equilibrium *P* is stable or unstable, the real part of the leading eigenvalue is on the order of $10^{-4}$. As such, the ribosome abundance in our model adjusts on an order of hours, rather than minutes, since it is dominated by the protein decay rate and the growth rate which are of the same order.

These results suggest that the delayed negative feedback in the control of ribosome abundance does not cause sustained oscillations within the range of biologically relevant parameters. This is caused primarily by two factors: the dilution by growth rate sets an upper bound on the length of destabilizing delays, and high stability of the ribsomal proteins causes low turnover of ribosomes in the model. This suggests that other control mechanisms like ribosome hibernation and deactivation (Prossliner et al. 2018) that operate on a much shorter time scale are essential to the rapid adjustment of the number of active ribosomes following a nutritional downshift. Therefore the experimental measurements in Figure 1 are likely caused by decaying oscillations in individual cells, but the attenuation rate of these oscillations is due to de-synchronization of the cell population.

This paper is organized as follows. Section 2 contains the derivation of the model. Section 3 states the results of our analysis, first for the ODE system and then for the full DDE system. The theorems describe the parameter regimes for which a single globally asymptotically stable equilibrium, *Q*, exists and those for which two equilibria, *Q* and *P*, exist and *P* becomes the locally asymptotically stable point. The transcritical bifurcation giving rise to *P* is given explicitly. Section 4 focuses on the equilibrium point *P*, showing that there are no steady-state bifurcations by which *P* can lose stability. We then turn to a numerical investigation of the DDE system, exploring the conditions under which *P* is de-stabilized through a Hopf bifurcation. An analysis of the Hopf points is also given in that section. Sections 5 and 6 contain the proofs of the results stated in Section 3. A final discussion and future work is contained in Section 7.

## Model Description and Assumptions

In this section, we develop a delay differential equation (DDE) model for ribosome abundance control. The starting point of our model is the earlier study (Shea et al. 2023) that developed an ordinary differential equation (ODE) model of ribosome abundance control in *E. coli*. We begin with the equations below.

1 $$\begin{aligned} \dot{R}&= \hat{\alpha }r p - GmR - \xi R, \nonumber \\ \dot{p}&= K G m R- \hat{\alpha } r p -\xi p, \nonumber \\ \dot{r}&= A - \hat{\alpha } r p-\beta r, \nonumber \\ \dot{m}&=f(p) - \frac{G}{\ell } mR -\beta m, \nonumber \\ \dot{n}&=I-\gamma n(A+B), \nonumber \\ \dot{g}&=H-\eta g, \end{aligned}$$

where

$$\begin{aligned} H&=\max (I_0-I,0),\\ A&= \left( A_{max} \frac{n}{\kappa _1 + n} + A_{0} \right) \frac{1}{\kappa _2 + g}, \\ B&= \left( B_{max} \frac{n}{\kappa _3 + n} + B_{0} \right) \frac{1}{\kappa _4 + g}. \end{aligned}$$

Here *R* is the concentration of free ribosomes, *p* stands for the concentration of ribosomal proteins, *m* represents the concentration of free ribosomal protein mRNA and *r* is the concentration of ribosomal RNA scaffolds (rRNA). A ribosome is assembled when ribosomal proteins bind to an rRNA. The variable *n* represents the concentration of free nucleoside triphosphate (NTP), the building block of both rRNA and mRNA, and *g* concentration of ppGpp, a molecule that contributes to ribosomal abundance control. The constant *I* models abundance of cellular resources. See (Shea et al. 2023) for full derivation of the model.

The model in (1) does not take into account delays which arise during each of the following processes: transcription of the rrn gene producing rRNA *r*, transcription of DNA to produce mRNA *m*, and translation of *m* producing *p*. We make the following observations:

1. Production of protein *p* at the current time depends on concentration of mRNA *m* and ribosomes *R*, $\tau _p$ units of time in the past. The time $\tau _p$ is the time of translation.
2. rRNA production has initiation rate $A(t-\tau _r)$, where $\tau _r$ is the time between initiation or transcription and release of rRNA.
3. mRNA production of *m* has initiation rate $B(t-\tau _m)$, where $\tau _m$ is the duration of transcription of gene for *m*.
4. Effect of *p* on suppression of free *m* production is mediated by preventing the translation of *m* into protein. This happens at the end of the transcription process and therefore we use *p*(*t*) rather than $t-\tau _m$ in the expression
  $$\begin{aligned} f(p) = \frac{B(t-\tau _m)}{\kappa + p(t)} . \end{aligned}$$

For this work, we have made the assumption that all the delayed processes have a constant delay. However, as was argued in Gedeon et al. (2022), these delays may depend on the physiological state of the cell and thus, may be in fact, state-dependent. Using the above observations, the model takes the form

2 $$\begin{aligned} \dot{R}&= \hat{\alpha } r p + e^{-\mu \tau _p}Gm(t-\tau _p)R(t-\tau _p) - GmR - (\xi + \mu )R, \nonumber \\ \dot{p}&= K G e^{-\mu \tau _p}m(t-\tau _p) R(t-\tau _p)- \hat{\alpha } r p - (\xi +\mu ) p, \nonumber \\ \dot{r}&= e^{-\mu \tau _r}A(t-\tau _r) - \hat{\alpha } r p-(\beta +\mu ) r, \nonumber \\ \dot{m}&=\frac{ e^{-\mu \tau _m}B(t-\tau _m)}{\kappa + p(t)} + G e^{-\mu \tau _p}m(t-\tau _p)R(t-\tau _p) - G mR -(\beta +\mu ) m, \nonumber \\ \dot{n}&=I-\gamma n(A+B), \nonumber \\ \dot{g}&=H-\eta g . \end{aligned}$$

Importantly, this system is not simply an ODE system (1) with delays inserted. Note that the terms with a delay are multiplied by a factor $e^{-\mu \tau }$, for some value of τ. This stems from the fact that the cell grows at a rate μ, and thus, its volume grows with time as $e^{\mu t}$. Therefore the concentration of a fixed number of molecules decreases at the rate $e^{-\mu t}$. This results in a decrease of the concentration of molecules (such as ribosomes) between the time τ units ago and the present time, when they re-enter their free pools. Observe, that the model (2) admits a negative feedback loop with delay, since *p* depends positively on $m(t-\tau _p)$, and *m*, in turn, depends negatively on *p*.

The next goal is to simplify the model by making the following modeling assumptions.

- There is a steady supply of nutrient I(t)=I and as a result the values of *n*(*t*), *g*(*t*) have achieved their steady state values.
- As a consequence, we assume that both A(t)=A and B(t)=B achieved constant values as well.
- We assume that the ribosomal scaffolds *r*(*t*) have reached a constant level $r(t) = r_0$.
- Using this steady state value $r_0$ we set $\alpha := \hat{\alpha } r_0$.
- Finally, to simplify the analysis we assume $\tau := \tau _p=\tau _r=\tau _m$.

Under these assumptions, the resulting system consists of three equations that track the evolution of the concentration of free ribosomes *R*(*t*), the concentration of ribosomal proteins *p*(*t*), and the concentration of free ribosomal protein mRNA *m*(*t*).

$$\begin{aligned} \dot{R}&= \alpha p + e^{-\mu \tau } Gm(t-\tau )R(t-\tau ) - GmR - (\xi +\mu ) R, \\ \dot{p}&= K G e^{-\mu \tau } m(t-\tau ) R(t-\tau )- \alpha p - (\xi +\mu ) p, \\ \dot{m}&=\frac{e^{-\mu \tau } B}{\kappa + p(t)} + e^{-\mu \tau } Gm(t-\tau )R(t-\tau ) - G mR -(\beta +\mu ) m. \end{aligned}$$

To simplify the notation, we define the following parameters,

$$\begin{aligned} \gamma _1 := \xi +\mu , \qquad \gamma _2 :=\alpha + \xi + \mu , \qquad \gamma _3 := \beta + \mu , \end{aligned}$$

to arrive at the delay model that is considered in the remainder of this work.

3 $$\begin{aligned} \dot{R}&= \alpha p + e^{-\mu \tau } Gm(t-\tau )R(t-\tau ) - GmR - \gamma _1 R, \nonumber \\ \dot{p}&= K G e^{-\mu \tau } m(t-\tau ) R(t-\tau ) - \gamma _2 p, \nonumber \\ \dot{m}&=\frac{e^{-\mu \tau } B}{\kappa + p(t)} + e^{-\mu \tau } Gm(t-\tau )R(t-\tau ) - G mR -\gamma _3 m. \end{aligned}$$

### Remark 1

Here we observe that not all five of the parameters of interest are independent. Specifically, one can make appropriate variable and parameter definitions so that the qualitative dynamics of the model is completely determined by four parameters related to those currently appearing in the system above. However, we choose to analyze the system in (3) in its current form in order to highlight the dependencies on half-saturation constant κ and on the other original parameters that have inherent biological interpretation.

## Summary of the results

This section contains a summary of results beginning with an analysis of both the DDE system and the corresponding ODE system obtained by setting the delay τ=0. In the second part, we summarize results of the numerical investigation of Hopf bifurcations that de-stabilize the interior equilibrium *P*.

### Equilibria of the ODE System

We begin by considering the ODE system where we set the delay τ=0 in system (3) as given below.

4 $$\begin{aligned} \dot{R}&= \alpha p - \gamma _1 R, \nonumber \\ \dot{p}&= KGmR -\gamma _2 p, \nonumber \\ \dot{m}&= \frac{ B}{\kappa + p} - \gamma _3 m, \end{aligned}$$

We proceed to show that the following results hold.

#### Theorem 1

Consider the system (4) and let $d:=B - \frac{\kappa \gamma _1\gamma _2\gamma _3}{KG\alpha }$. Then,

- When d<0 then the equilibrium
  $$\begin{aligned} Q:= \left( 0,0, \frac{ B}{\kappa \gamma _3} \right) \end{aligned}$$
  is the only equilibrium in $R_0^{3+}$ and *Q* is globally asymptotically stable.
- When d>0 then there are two equilibria *Q* and $P=(R^*,p^*,m_P^*)$ with $R^*>0$, $p^*>0$, $m_P^*>0$. In addition, *P* is locally asymptotically stable for all values of d>0.
- When d=0 the equilibria *Q* and *P* coincide and undergo a transcritical bifurcation.

Theorem 1 recapitulates the qualitative dichotomy for the larger ODE model (1) that was studied in Shea et al. (2023). When d<0 the system stabilizes at *Q*, where the concentrations of free ribosomes and ribosomal proteins are zero, signifying that all ribosomes are engaged in translation of ribosomal proteins and all ribosomal proteins are immediately assembled into ribosomes. This state corresponds to a nutritional state with limited resources since no other proteins apart from ribosomal proteins are being produced. When d>0, the system exhibits two equilibria: the trivial equilibrium *Q* and an asymptotically stable equilibrium *P*, where all components are positive. It supports positive concentrations of free ribosomal proteins, free ribosomes and mRNA, which corresponds to state where cell has extra capacity to translate proteins other than the ribosomal proteins. Hence, Theorem 1 affirms that the simplifying assumptions that lead from (1) to (4) did not change the model’s dynamics, and the proof of Theorem 1 is given in Section 5.

### Equilibria of the DDE System

Consider the DDE system (3) with delay τ>0. We have the following results.

#### Theorem 2

Consider the system (3) and let $d:=B - \frac{\kappa \gamma _1\gamma _2\gamma _3}{KG\alpha }$. Then

- When d<0 then the equilibrium
  5 $$\begin{aligned} Q:= \left( 0,0, \frac{e^{-\mu \tau } B}{\kappa \gamma _3} \right) \end{aligned}$$
  is the only non-negative equilibrium and is locally asymptotically stable.
- When d>0, there exists $\tau _{crit}>0$ such that for $\tau \in (0,\tau _{crit})$, there exist two equilibria, *Q* as given in (5), which is unstable, and $P(\tau )=(R^*(\tau ),p^*(\tau ),m_P^*(\tau ))$ with $R^*>0$, $p^*>0$, $m_P^*>0$.
- When d=0 the equilibria *Q* and *P*(0) coincide.
- When d>0 and $\tau = \tau _{crit}$, *Q* and $P(\tau _{crit})$ coincide.
- When d>0, there exists σ such that 0<σ<1 and the equilibrium P(τ) is asymptotically stable when $\tau \in (0, \sigma ) \cup ((1-\sigma )\tau _{crit}, \tau _{crit}$). That is, P(τ) remains stable when the delay τ is sufficiently close to either the critical value $\tau _{crit}$ or to 0.

The proof of the Theorem 2 can be found in Section 6.

#### Remark 2

The most important and perhaps surprising observation is that the non-negative equilibrium *P* not only loses stability with increasing delay τ as can be expected for a system with delayed negative feedback, but that the equilibrium *P* itself depends on τ in such a way that for large enough τ, the equilibrium *P* exits the biologically feasible region. Biological explanation of this phenomena stems from the fact that when the delay is too long, the cell growth dilutes the ribosomes too much for them to sustain the cell growth. As we will see in (40), the delay value $\tau _{crit}$ at which the non-negative equilibrium *P* exits the nonnegative orthant is inversely proportional to cell growth rate μ. The existence of an upper limit $\tau _{crit}$ makes the question of whether increased delay can cause instability more interesting, since this instability must happen within a limited window $\tau \in (0,\tau _{crit}).$

Theorem 2 shows that there is a region of the feasible parameter space $\mathcal {R}$, given by d>0, where the equilibrium *P* exists for sufficiently small $\tau \in (0, \tau _{crit}).$ We now investigate whether there is a subset of these parameters where for some $\tau \in (0, \tau _{crit})$, *P* loses stability by a Hopf bifurcation.

As detailed in the next section, we sample 93, 750 parameter combinations uniformly from $\mathcal {R}\times T\times \zeta$, and for each combination, we compute the five eigenvalues with the largest real part. We find that *P* remains stable for the entire range $\tau \in (0, \tau _{crit})$ at all these samples.

**Fig. 2:**
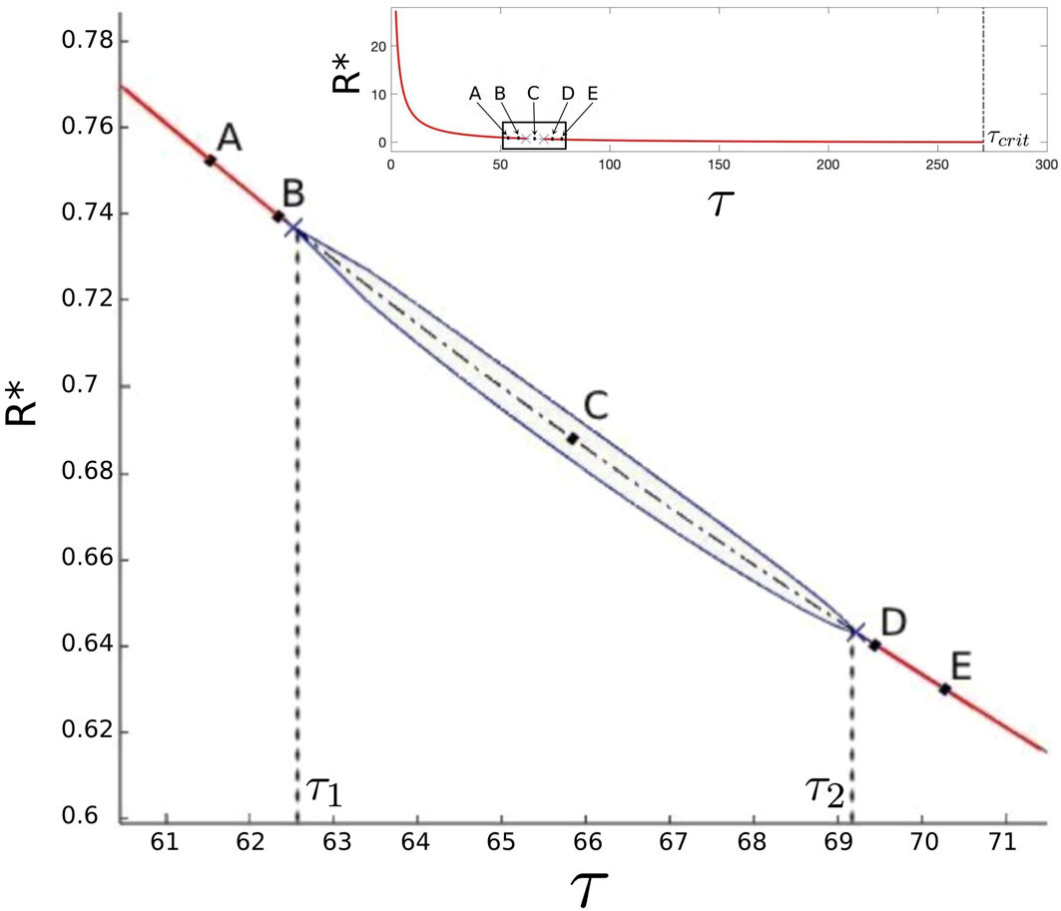
Behavior of the $R^*(\tau )$ component of the equilibrium $P(\tau )=(R^*(\tau ),p^*(\tau ),m^*(\tau ))$ when d>0 and $\tau \in (0,\tau _{crit})$. As is visible in the inset graph, $R^*(\tau ) \rightarrow 0$ as $\tau \rightarrow \tau _{crit}$. Here, for the fixed parameters $\alpha =0.9729, K=0.0650, B=0.45, \kappa =92.3346, G=0.9602, \gamma _1=0.02291, \gamma _2=0.9958, \gamma _3=0.00251$, we calculated $\tau _{crit} = 270.8770$ and $R^*(\tau _{crit}) = 4.6559 \times 10^{-9}$. The enlarged panel shows the $R^*$ projection of the stable periodic orbit between the Hopf bifurcations at $\tau _1 = 62.5054$ and $\tau _2 = 69.1513$. For the components $p^*(\tau )$ and $m^*(\tau )$, the behavior is similar. At $\tau _1 = 62.5054$ the equilibrium P(τ) undergoes a Hopf bifurcation and becomes unstable, while at $\tau _2 = 69.1513$ the equilibrium P(τ) regains stability

We repeat this calculation with the value of the protein decay rate ξ replaced by

$$\begin{aligned} \bar{\xi } := 100 \xi , \end{aligned}$$

which corresponds to a protein half-life of about 0.5 minutes. With this change, for the 93, 750 combinations in $\mathcal {R}\times T\times \bar{\zeta }$ we find 66 parameter combinations for which a (nonempty) range of values of τ has a leading pair of imaginary eigenvalues with positive real part. To elucidate the structure of the set of parameters where *P* is unstable, we perform a continuation analysis in the parameter space. We find that *P* is unstable for $\tau \in (\tau _1, \tau _2)$, which is bounded by a pair of Hopf bifurcations at $\tau _1$ and $\tau _2$ as shown in Figure 2. While at $\tau _1$ the leading pair of complex eigenvalues crosses the imaginary axis from left to right, and at $\tau _2$ it crosses back to left half-plane, restoring the stability of *P*. This is consistent with Theorem 2. We verify that the Hopf bifurcations at $\tau _1$ and $\tau _2$ are indeed connected by performing two parameter continuation, justifying the name *Hopf pair* for $[\tau _1, \tau _2]$. We detected no additional Hopf bifurcations at any $\tau \subset [\tau _1, \tau _2]$ in our samples within the parameter range $\mathcal {R}\times T\times \bar{\zeta }$.

We observe the following results from the analysis of the linearization of P(τ) at points A, B, C, D, and E as labelled in Figure 2. The leading real eigenvalue shifts when the system moves between A and B, resulting in the complex pair being dominant. Since all eigenvalues remain in the left half plane, the system experiences damped oscillations and remains stable. The real part of the leading complex pair becomes positive as point B is crossed, making *P* unstable via a Hopf bifurcation. At the point C, the equilibrium *P* is unstable and there is a stable periodic orbit in the unstable manifold of *P*. Equilibrium *P* regains its stability at the point D when the real part of the complex pair turns negative, and finally, between D and E the leading complex pair loses dominance and a negative real eigenvalue again becomes the dominant eigenvalue.

## Destabilizing *P* in the DDE system

Since the calculations in this section are concerned exclusively with the equilibrium *P*, we simplify the notation $m^*_P$ to simply $m^*$ for brevity. In later sections, we return to the subscript notation when it is important to distinguish between *P* and *Q*. To find conditions when the equilibrium *P* can be destabilized, we first linearize the system (3) at $P=(R^*,p^*,m^*)$.

We substitute,

$$ R=R^*+x e^{\lambda t}, \quad p=p^*+y e^{\lambda t}, \quad m=m^*+z e^{\lambda t}$$

into (3), and after some algebraic manipulations, we arrive at the following linear system:

6 $$\begin{aligned} \lambda x&= \alpha y + G (e^{-(\mu +\lambda ) \tau }-1)(m^*x +R^* z) -\gamma _1 x, \end{aligned}$$

7 $$\begin{aligned} \lambda y&= K G e^{-(\mu +\lambda ) \tau }(m^*x +R^* z) -\gamma _2 y, \end{aligned}$$

8 $$\begin{aligned} \lambda z&= -\frac{e^{-\mu \tau } B}{(\kappa + p^*)^2} y + G (e^{-(\mu +\lambda ) \tau }-1)(m^*x +R^* z) -\gamma _3 z. \end{aligned}$$

System (6–8) admits a non-trivial solution if and only if

9 $$\begin{aligned} p(\lambda ,\tau ):=\det \begin{bmatrix} \lambda +\gamma _1+Gm^*(1-L M) & -\alpha & GR^*(1-L M) & \\ -K G m^* L M & \lambda +\gamma _2 & -K G R^* LM \\ Gm^*(1-LM) & M H & \lambda +\gamma _3+ GR^*(1-LM) \end{bmatrix}=0, \end{aligned}$$

where we let

$$ L= e^{-\lambda \tau },\quad M=e^{-\mu \tau } \in (0,1] ,\quad H = \frac{B}{(\kappa + p^*)^2}.$$

By subtracting row 1 from row 3 in (9), we find that

$$ p(\lambda ,\tau )=\det \begin{bmatrix} \lambda +\gamma _1+Gm^*(1-L M) & -\alpha & GR^*(1-L M) & \\ -K G m^* L M & \lambda +\gamma _2 & -K G R^* LM \\ -(\lambda +\gamma _1) & M H+\alpha & \lambda +\gamma _3 \end{bmatrix}.$$

Expanding along the bottom row, we find that

$$\begin{aligned} p(\lambda ,\tau )&= -(\lambda +\gamma _1)[ \alpha K GM LR^* - (\lambda +\gamma _2)GR^*(1-LM)]\\&-(MH+\alpha )[-K GM LR^* (\lambda +\gamma _1+G m^*(1-LM))+KGM Lm^* GR^*(1-LM)\\&+ (\lambda +\gamma _3)[(\lambda +\gamma _2)(\lambda +\gamma _1+G m^*(1-LM))-\alpha KGMLm^*]. \end{aligned}$$

$$\begin{aligned} p(\lambda ,\tau )&= -(\lambda +\gamma _1)[ \alpha K GM LR^* - (\lambda +\gamma _2)GR^*(1-LM)]\\&+K GM LR^* (\lambda +\gamma _1)(MH+\alpha )\\&+ (\lambda +\gamma _3)[(\lambda +\gamma _2)(\lambda +\gamma _1+G m^*(1-LM))-\alpha KGMLm^*]. \end{aligned}$$

$$\begin{aligned} p(\lambda ,\tau )&= GR^*(\lambda +\gamma _1)[ (\lambda +\gamma _2)(1-LM)+ KM^2 HL]\\&+ (\lambda +\gamma _3)[(\lambda +\gamma _2)(\lambda +\gamma _1+G m^*(1-LM))-(\gamma _1+ G m^* (1-M))\gamma _2 L], \end{aligned}$$

where we use the equilibrium relation

$$ (\gamma _1+ G m^* (1-M))\gamma _2=\alpha KG m^* M$$

from system (3).

Finally, we arrive at

10 $$\begin{aligned} p(\lambda ,\tau )&= GR^*(\lambda +\gamma _1)[ (\lambda +\gamma _2)(1-LM)+ KM^2 HL] \nonumber \\&+ (\lambda +\gamma _3)[\lambda ^2 +\lambda (\gamma _2+\gamma _1+G m^*(1-LM))+\gamma _2(\gamma _1+ G m^*)(1-L)]. \end{aligned}$$

Substituting λ=0 and L=1 into (10), we readily find that

$$ p(0,\tau )= G R^* \gamma _1[(1-M) \gamma _2 +M^2 H K] ,$$

which is greater than zero whenever $R^*>0$ which is equivalent to P≠Q. This proves the following Lemma.

### Lemma 1

The value λ=0 is not an eigenvalue of (6–8) for any $\tau \in (0,\tau _{crit})$. Therefore, there are no steady-state bifurcations with $\tau \in (0,\tau _{crit})$ that involve *P*.

This confirms our earlier finding that *P* is the unique positive equilibrium, whenever it exists. Having established that *P* cannot lose stability through a steady-state bifurcation, we now turn to considering Hopf bifurcations.

### Numerical Investigation of Hopf Bifurcations at *P*

For the numerical investigation of the equilibrium *P* in the DDE system (3), we use the sampled parameter space $\mathcal {R}\times T\times \zeta$ and calculate the eigenvalues of (6–8) corresponding to each parameter combination. These numerical computations show that, for all parameter combinations sampled, the leading eigenvalues have a real part on the order of $10^{-4}$. This slow dynamics is explained by the fact that both the growth rate μ and the protein decay rate ξ are of this order. We categorize these eigenvalues into two cases as follows. *Case 1:* The leading eigenvalue is real. *Case 2:* The leading eigenvalues form a complex conjugate pair. Examining the parameters falling into *Case 2*, we observe that in the sampled space $\mathcal {R}\times T\times \zeta$, the eigenvalues always remain in the left half plane. Thus, we are unable to detect any Hopf point in $\mathcal {R}\times T\times \zeta$ (i.e. for the biologically relevant value of ξ).

#### Hopf Bifurcations become possible at faster protein decay rate

When ξ is increased to $\bar{\xi }=100\xi$, we identified 66 parameter combinations in $\mathcal {R}\times T\times \bar{\zeta }$ that belong to *Case 2*, all of which have the leading complex conjugate pair in the positive half-plane. However, the real part of these conjugate pairs stays on the order of $10^{-4}$ despite the increased decay rate since the growth rate is still of order $10^{-4}$. Then we performed a one-parameter continuation in both directions along τ for these 66 parameter combinations, while holding the values of the parameters in $\mathcal {R} \times \bar{\zeta }$ fixed, to find nearby Hopf bifurcations. Starting from each such Hopf bifurcation, a two-parameter continuation is then performed by selecting two parameters from the six-dimensional parameter space consisting of five parameters comprising $\mathcal {R}$ and τ, while the values of the parameters in $\bar{\zeta }$ are held fixed. The continuation process extends a Hopf branch (Sieber et al. 2017, 24) by combining predictions and corrections. Each new point is predicted using secant prediction (Gu et al. 2010) based on previously computed points with an appropriate step length.

**Fig. 3:**
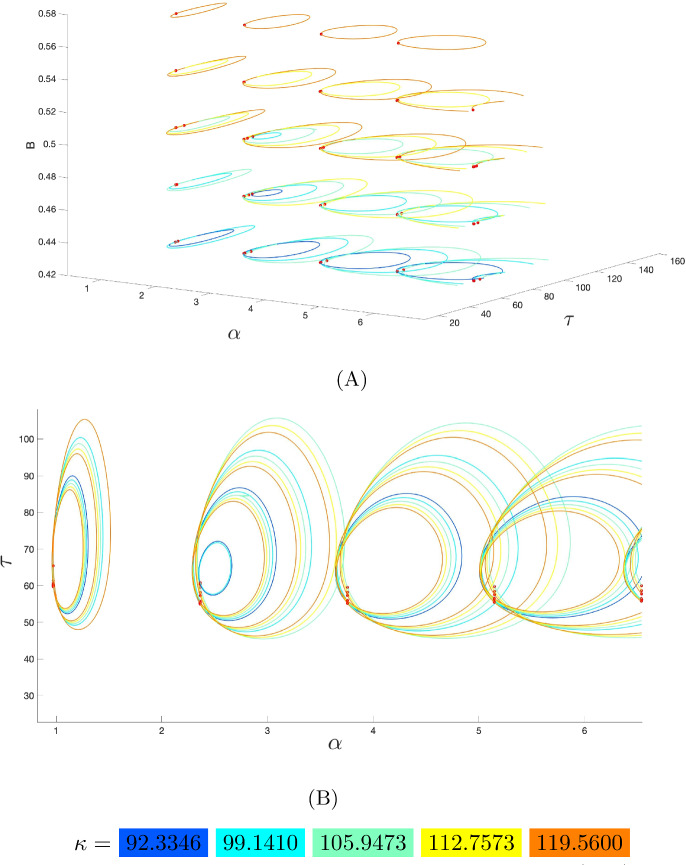
The two-parameter continuations of Hopf bifurcation in $(\alpha ,\tau )$ corresponding to the 66 parameter combinations are illustrated in both panels (A) and (B). Panel (B) is a projection of the information in Panel (A) onto the $(\alpha ,\tau )$ - plane. The parameters *K* and *G* are fixed for these simulations, the five distinct colors indicate different values of parameter κ in each continuation. As can be observed, some of the continuation curves do not form a complete curve when they approach the upper bound of the α range since the DDE Biftool terminates the continuation curves as they approach the prespecified parameter bounds

We observe that Theorem 2(e) guarantees that there is always a pair of Hopf bifurcations at $\tau _1 < \tau _2$ for these 66 parameter combinations. These two Hopf bifurcations, forming a Hopf pair, are connected in the six-dimensional parameter space by a curve of Hopf bifurcations. For each parameter combination that admits the Hopf bifurcations, the Hopf pair appears to be confined to values for τ between 45 and 105 seconds. Figure 3 illustrates the results of the two-parameter continuations of Hopf bifurcation with different pairs of parameters.

#### Additional Hopf bifurcations

Once the 66 parameter combinations in $\mathcal {R}\times T\times \bar{\zeta }$ at which Hopf pairs $\tau _1<\tau _2$ occur were determined, we sampled 10 values of τ in each interval $(\tau _1,\tau _2)$ respectively. The corresponding eigenvalues were calculated and categorized into *Case 1* and *Case 2* as defined at the beginning of Section 4.1. In each interval $(\tau _1,\tau _2)$, we observed that all of these eigenvalues fall under *Case 1*. By Lemma 1 this implies that no further eigenvalues cross into the positive half-plane when $\tau \in (\tau _1,\tau _2)$.

### Hopf Point Analysis

Results in the previous section show that Hopf pairs exists in a certain region of the parameter space defined by the biological problem under consideration. In this section, we move a step further where we seek to understand if the linearization at *P* can exhibit additional Hopf points for parameter choices that lie outside the biologically feasible region. Mathematically we seek to identify conditions under which a second pair, or even a third pair of eigenvalues, cross into the positive half-plane, respectively. To answer this question, we turn to Hopf point analysis.

#### Definition 1

* A Hopf point* is the value of the parameter τ such that the linearization (6–8) has purely complex pairs of eigenvalues. * A Hopf pair* is a pair of Hopf bifurcations such that the periodic orbits emanating from them, are connected by a continuous branch of periodic orbits. Stability of these periodic orbits may change along the branch.

To investigate the existence of Hopf points, we represent the characteristic polynomial at *P* as

11 $$\begin{aligned} p(\lambda ,\tau ) = \mathcal {A}(\lambda ) L + \mathcal {B}(\lambda ), \end{aligned}$$

where

$$\begin{aligned} \mathcal {A}(\lambda )&= GR^*M(\lambda +\gamma _1)[KMH-(\lambda +\gamma _2)]-(\lambda +\gamma _3)[MG m^* \lambda +\gamma _2(\gamma _1+G m^*)]\\ \mathcal {B}(\lambda )&= (\lambda +\gamma _2)[GR^*(\lambda +\gamma _1)+(\lambda +\gamma _3)(\lambda +\gamma _1+G m^*)]. \end{aligned}$$

In particular, we have that

$$\begin{aligned} \mathcal {A}(0)&= GR^*M\gamma _1[KMH-\gamma _2]-\gamma _3\gamma _2(\gamma _1+G m^*),\\ \mathcal {B}(0)&= \gamma _2[GR^*\gamma _1+\gamma _3(\gamma _1+G m^*)]>0,\\ p(0,\tau )&= \mathcal {A}(0)+\mathcal {B}(0) >0. \end{aligned}$$

Due to the equilibrium relations from system in (3),

$$KMGR^* m^*=\gamma _2p^*, \quad \frac{M B}{(\kappa +p^*)m^*}=\gamma _3 +(1-M) GR^*,$$

and inequality $\frac{p^*}{\kappa +p^*}<1$, we have that

$$ GR^*M\gamma _1KMH =\gamma _1 \gamma _2 \frac{M H p^*}{m^*} < \gamma _1 \gamma _2 \frac{M B}{(\kappa +p^*)m^*}=\gamma _1 \gamma _2(\gamma _3+(1-M) GR^*).$$

Consequently,

$$\begin{aligned} \mathcal {A}(0)&< \gamma _1 \gamma _2(\gamma _3+(1-M) GR^*) - \gamma _1 \gamma _2 GR^*M -\gamma _3\gamma _2(\gamma _1+G m^*)\\&= \gamma _1 \gamma _2 (1-2 M) GR^* - \gamma _2 \gamma _3 Gm^*< \gamma _1 \gamma _2 GR^* < \mathcal {B}(0). \end{aligned}$$

Since $-\mathcal {B}(0)< \mathcal {A}(0)< \mathcal {B}(0)$, we conclude that

$$ |\mathcal {A}(0)|< |\mathcal {B}(0)| \qquad \text{ and } \text{ therefore } \qquad \left| \frac{\mathcal {B}(0)}{\mathcal {A}(0)} \right| >1.$$

We note that $\mathcal {A}(\lambda )$ is quadratic in λ, while $\mathcal {B}(\lambda )$ is cubic in λ. Consequently,

$$ \lim _{|\lambda | \rightarrow \infty } \left| \frac{\mathcal {B}(\lambda )}{\mathcal {A}(\lambda )} \right| =+\infty .$$

In particular, if one sets $\lambda := i \omega$, it follows that $|\mathcal {B(i \omega )}/\mathcal {A}(i \omega )|>1$ for all |ω|>>1 as well as for all |ω|<<1. Finally, we observe that

$$D(w):= \left| \frac{\mathcal {B}(i \omega )}{\mathcal {A}(i \omega )} \right| =1 \quad \Longleftrightarrow \quad \phi (\omega ):=|\mathcal {B}(i \omega )|^2-|\mathcal {A}(i \omega )|^2=0,$$

where $\phi (\omega )$ is an even polynomial of degree 6 with $\phi (\omega )>0$ for all |ω|>>1 as well as for all |ω|<<1. Hence there are only two (generic) possibilities:

*Case (I):* Either $\phi (\omega )>0$ for all $\omega \in \mathbb R'$; or

*Case (II):* there exist at least two values $0<\omega _1 < \omega _2$, such that $\phi (\omega )<0$ for $\omega \in (\omega _1, \omega _2).$

#### Remark 3

Since $\phi (\omega )$ is a polynomial of degree 6 there can be at most 6 roots

$$\begin{aligned} \omega _1<\omega _2< \ldots < \omega _6. \end{aligned}$$

Also, since the polynomial $\phi (\omega )$ is even, its roots must be symmetric about the origin. So there can be 0,2,4 or 6 real roots (counting their multiplicities). If $\phi (\omega )$ had six real roots, then there would be three real roots on each side of ω=0 and this would imply $\phi (0) \phi (\infty )<0$, a contradiction. Hence, six real roots is an impossibility. If $\phi (\omega )$ had two simple real roots, again we would have $\phi (0) \phi (\infty )<0$, another impossibility. Therefore, the only two possibilities are 0 real roots (Case I), or 4 real roots (2 on each side of ω=0) (Case II). However, our numerical simulations across the biologically feasible parameters found no roots in $\mathcal {R}\times T\times \zeta$. In the space $\mathcal {R}\times T\times \bar{\zeta }$ we found either no roots or two roots $\omega _1 <\omega _2$.

We now continue our analysis in the following way. With all other parameters fixed, the equilibrium P(τ) depends only on the delay τ through the value of $M = e^{-\mu \tau }$. We start by fixing the value of M∈(0,1). Then the coordinates of the equilibrium are independent of the delay τ, as long as the value of μ is adjusted to keep *M* constant. We look for solutions of $p(i\omega ,\tau ) =0$, defined in (11), in the equivalent form $L =e^{-i\omega \tau } = - \mathcal {B(i \omega )}/\mathcal {A(i \omega )}.$ Observe, that to have $\omega \tau \in \mathbb R$, we must have

$$ |e^{-i\omega \tau }| = | \mathcal {B(i \omega )}/\mathcal {A(i \omega )}|=1.$$

However, in the *Case (I)* above, $\phi (\omega ) >0$ and hence $D(\omega ) =| \mathcal {B(i \omega )}/\mathcal {A(i \omega )}| >1$ and no solutions with $\omega \tau \in \mathbb R$ exist. Consequently, Hopf point is impossible and the positive equilibrium will remain locally stable.

On the other hand, in the *Case (II)* above, there are at least two distinct values $0<\omega _1<\omega _2$ such that

$$ e^{-i \omega _k \tau _k}=- \mathcal {B(i \omega _k)}/\mathcal {A(i \omega _k)} = e^{i \theta _k}, \quad k=1,2,$$

for some $\theta _k \in \mathbb R$. Then

12 $$\begin{aligned} \tau ^n_k(M)=\frac{-\theta _k + 2 \pi n}{\omega _k},\ n \in {\mathbb Z} \end{aligned}$$

gives all values of the delay corresponding to the Hopf points $p(i \omega _k,\tau _k)=0.$

For two solutions $\omega _1(M)$ and $\omega _2(M)$, the formula (12) produces two sequences of τ values

$$\begin{aligned} &  \tau _1^1(M)< \tau _1^2 (M)< \tau _1^3(M)< \ldots \\ &  \tau _2^1(M)< \tau _2^2(M)< \tau _2^3(M) < \ldots . \end{aligned}$$

Finally, using the formula $M = e^{-\mu \tau }$ using the value of the fixed *M*, we find for each $\tau _k^n$ the corresponding value of μ

$$\begin{aligned} \mu _k^n(M) := - \frac{\ln M}{\tau _k^n(M)}. \end{aligned}$$

**Fig. 4:**
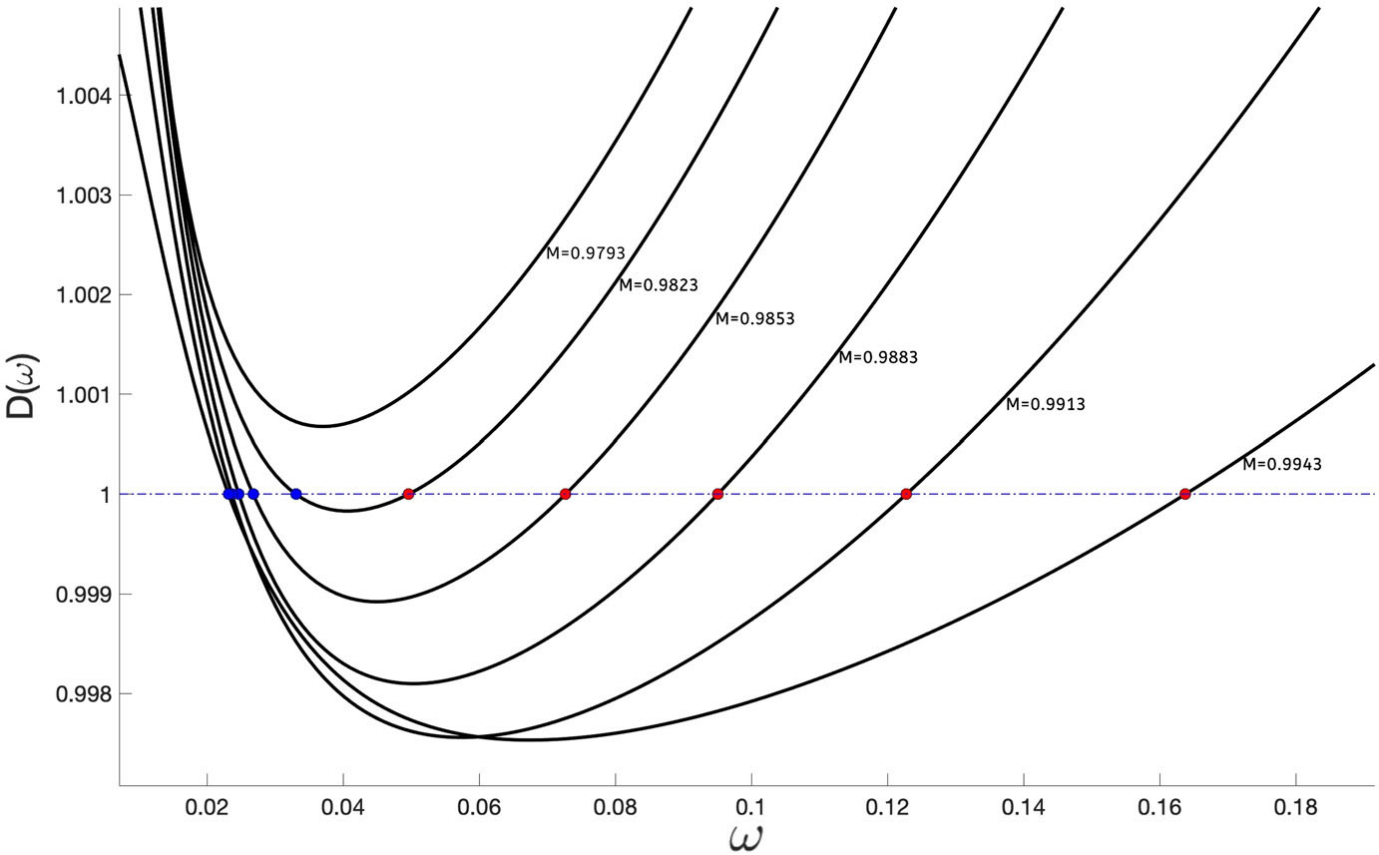
$D(\omega )=\left| \frac{\mathcal {B}(i \omega )}{\mathcal {A}(i \omega )} \right|$ as a function of *M* for the fixed parameters $\alpha =0.9729, K=0.0650, B=0.45, \kappa =92.3346, G=0.9602, \gamma _1=0.02291, \gamma _2=0.9958, \gamma _3=0.00251$. There are no roots for D(ω) for M=0.9793 while there are exactly two roots for all other M∈(0.9817,1) namely $\omega _1(M)$ in blue and $\omega _2(M)$ in red. It is clear that $\omega _2(M)$ grows in size while $\omega _1(M)$ remains small

We numerically investigate the structure of the Hopf points $(\tau _k^j, \mu _k^j)$ for k=1,2 and j=1,2,3 as a function of two parameters μ and τ. Notably, this structure depends on the choice of other parameters in the parameter space $\mathcal {R}\times \bar{\zeta }$. As described above, we first sampled M∈(0,1) to find values of *M* for which the function $\phi (\omega )$ falls into *Case (II)* and has two zeroes $\omega _1(M)< \omega _2(M)$. We find that *Case (II)* happens for M∈(0.9817,1) (see Figure 4).

We observed that at value M=0.9817 two roots coincide $\omega _1=\omega _2$.

For this value of *M*,

$$\begin{aligned} \tau _1^j(M) = \tau _2^j (M) \quad \text{ for } \text{ all } j = 1,2,3,\ldots . \end{aligned}$$

**Fig. 5:**
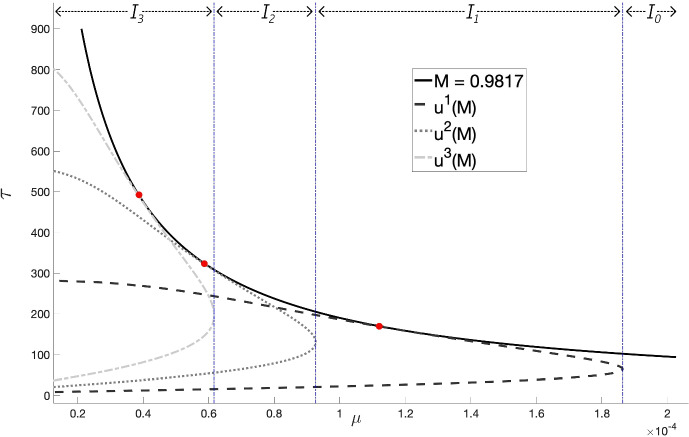
The $(\mu ,\tau )$ plane illustrates the curves $u^j(M)$ for j=1,2,3. Each $u^j(M)$ is a union of $c_2^j(M)$ and $c_1^j(M)$ for j=1,2,3. Here, $c_k^j(M) := (\tau _k^j, \mu _k^j)$ for k=1,2 and j=1,2,3. The $\tau _k^j(M)$ curves correspond to the solutions $\omega _1(M)$ and $\omega _2(M)$ obtained by solving the function D(ω)=1. M=0.9817 curve intersects each $u^j(M)$ curve at the red point where $c_2^j(M)$ and $c_1^j(M)$ converge for *j* = 1, 2, 3. When $\mu \in I_0$ that there are no Hopf points for $\tau \in (0, \tau _{crit})$, while in intervals $I_j$ there are 2*j* Hopf points in $\tau \in (0, \tau _{crit})$

We graph the function $0.9817 = e^{-\mu \tau }$ in Figure 5 as a solid black curve. We then use the two parameter continuation in DDE Biftool to continue Hopf points $c_k^j(M) := (\tau _k^j, \mu _k^j)$ for k=1,2 and j=1,2,3.

The dashed black curve $u^1(M)$ in Figure 5 is the union of $c_2^1(M)$ and $c_1^1(M)$; these two curves meet on the curve $0.9817 = e^{-\mu \tau }$. The intersection of $u^1(M)$ and $0.9817 = e^{-\mu \tau }$ is marked as a red dot. The part above the intersection is $c_1^1(M),$ while the part below the intersection is $c_2^1(M)$. Similar comments apply to dotted dark gray curve $u^2(M)$, which is the union of $c_2^2(M)$ (bottom part) and $c_1^2(M)$ (top part), and dashed-dotted light gray curve $u^3(M)$ which is the union of $c_2^3(M)$ (bottom part) and $c_1^3(M)$ (top part).

The vertical lines in Figure 5 divide the range of μ into non-overlapping open intervals

$$\begin{aligned} ... I_3< I_2<I_1< I_0, \end{aligned}$$

such that for $\mu \in I_j, j=0,1,2,3$ there are 2*j* Hopf points which correspond to intersection of μ=const with curves $c_k^j$. Note that we only computed curves $u^j(M)$ for j=1,2,3. In our sampling of 93,750 combinations of the parameter space $\mathcal {R} \times T \times \bar{\zeta }$, we found 66 combinations at which we observed a single Hopf pair. In the context of Figure 5, these are the combinations where the value of μ=0.00018 falls within the interval $I_1$; for the remaining parameter combinations, the value of μ falls within the interval $I_0$.

Let $$u^j(M) := c_2^j(M)\cup c_1^j(M)$$ and

$$ \mu _{\max }^j := \max _{(\mu ,\tau ) \in u^j(M)} \mu (M). $$

We conjecture that for all j=1,2,…

$$\begin{aligned} \mu _1(M)> \mu _2(M)> \mu _3(M)> \mu _4(M) > \ldots , \end{aligned}$$

and therefore there are intervals $I_j:=(\mu _{\max }^{j+1},\mu _{\max }^j)$ such that

$$\begin{aligned} ...<I_j< \ldots< I_4<I_3< I_2<I_1< I_0 \end{aligned}$$

holds for all *j*.

Consequently, we have the following conjecture.

#### Conjecture 1

For any parameter *p* for which D(ω)=1 has two roots $\omega _1 < \omega _2$, if $\mu \in I_k$ there are 2*k* Hopf points at *P*.

**Fig. 6:**
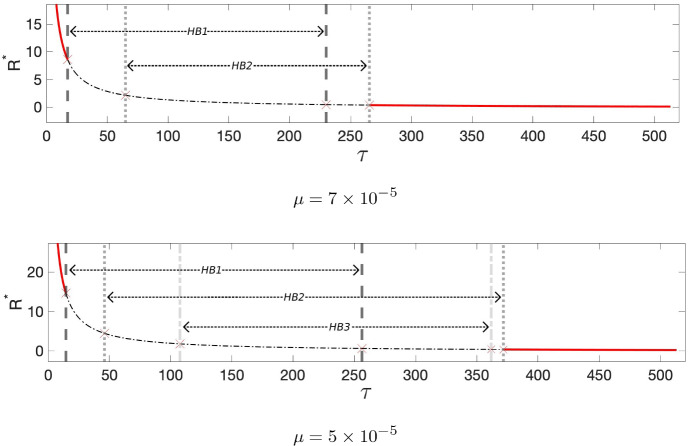
Behavior of the $R^*(\tau )$ component of the equilibrium $P(\tau )=(R^*(\tau ),p^*(\tau ),m^*(\tau ))$ with the following fixed parameters: $\alpha =0.9729, K=0.0650, B=0.45, \kappa =92.3346, G=0.9602$. For $\mu = 7 \times 10^{-5}$, $\gamma _1 =0.02337, \gamma _2 = 0.99627, \gamma _3=0.00240$. For $\mu = 5 \times 10^{-5}$, $\gamma _1 =0.02335, \gamma _2 = 0.99625, \gamma _3=0.00238$. We observe four Hopf points for $\mu = 7 \times 10^{-5}$ $\in I_2$ and six Hopf points for $\mu = 5 \times 10^{-5}$ $\in I_3$. In the first panel, there are two intervals labeled as HB1, and HB2 respectively for $\tau \in [17.3793, 230.1908]$ and $\tau \in [64.8907, 265.3908]$. In the second panel, there are three intervals labeled as HB1, HB2, and HB3 respectively for $\tau \in [14.4439, 256.0064]$, $\tau \in [45.9059, 371.6054]$, and $\tau \in [107.6064, 361.7075]$. For the components $p^*(\tau )$ and $m^*(\tau )$, the behavior is similar

**Fig. 7:**
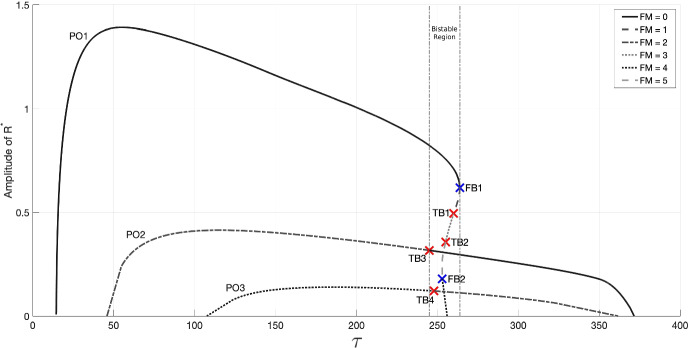
Stability of periodic orbits with $\mu = 5\times 10^{-5}$ for the $R^*(\tau )$ component of the equilibrium $P(\tau )=(R^*(\tau ),p^*(\tau ),m^*(\tau ))$. For the components $p^*(\tau )$ and $m^*(\tau )$, the behavior is similar. See the text for detailed explanation of the stability of branches and observed bifurcations (crosses). The line types describe the number of unstable Floquet multipliers of the periodic orbits (see the legend)

In principle, the 2*k* Hopf points can be arranged in different ways. In Figure 5 there are either zero or two intersections of μ=const and curve $u^j(M)$. In case there are two, let $h^j_f$ be the lower intersection and $h^j_b$ be the higher intersection. Note that at $\tau =h^j_f$ the complex pair of eigenvalues crosses from the negative half-plane to the right positive half-plane and at $\tau =h^j_b$ from right to left. Let HBj$:=[h^j_f, h^j_b]$ be the interval of the parameter τ when a particular pair of eigenvalues associated with the curve $u^j(M)$ is in the positive half-plane. Since we were able to continue periodic orbits between each of the intervals HBj in Figure 6 using the DDE Biftool, all of these τ pairs can be considered as Hopf pairs; therefore, these Hopf points are actually Hopf bifurcations. The stability of the periodic orbits for $\mu = 5\times 10^{-5}$ is shown in Figure 7. There are three distinct Hopf pairs that correspond to three periodic orbits. The first periodic orbit PO1, is stable for $\tau \in [14.4439, 263.8305]$ where it undergoes a *fold bifurcation* (marked as FB1) first. Following this bifurcation, the periodic orbit on the PO1 branch has one unstable Floquet multiplier. The PO1 branch then undergoes two $\textit{torus bifurcations}$, denoted TB1 at τ=260.0803 and TB2 at τ=255.1805, resulting in a periodic orbit with 3 and 5 unstable Floquet multipliers, respectively. Finally, before the PO1 branch terminates in a Hopf bifurcation at τ=256.0064, it undergoes a second fold bifurcation FB2 at τ=252.9600, resulting in 4 unstable Floquet multipliers. The second bend in the PO1 branch is not easily visible in Figure 7 because the periodic orbit emanating from the right end of the interval HB1 grows in amplitude rapidly in a very short interval of τ.

The second periodic orbit PO2 exists for τ in the interval HB2 =[45.9059,371.6054]. The periodic orbit on the branch PO2 has 2 unstable Floquet multipliers before a bifurcation at τ=245.0181 (marked as TB3), and it becomes stable after that. The third periodic orbit PO3 exists in the interval HB3 =[107.6064,361.7075]. Since it has 4 unstable Floquet multipliers before a bifurcation at τ=247.8711 (marked as TB4) and 2 unstable Floquet multipliers after that, PO3 is always unstable. Since two Floquet multipliers cross the unit circle at TB3 and TB4, these points also correspond to torus bifurcations. Since the periodic orbit PO2 is stable to the right of TB3, it is possible that to the left of TB3 there may be a stable invariant torus.

**Fig. 8:**
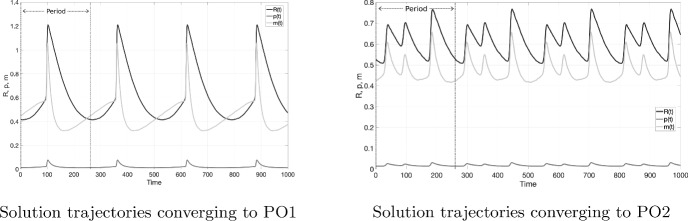
Behavior of the solutions (*R*(*t*), *p*(*t*), *m*(*t*)) within the bistable region with a delay τ=260. The left panel illustrates solution converging to periodic orbit PO1, while the right panel shows solution converging to the periodic orbit PO2

Importantly, since PO1 and PO2 are both stable for $\tau \in [245.0181, 263.8305]$, in this region we have a coexistence of two stable periodic orbits, see Figure 7. For τ within this region, there are initial conditions that converge to PO1 and other initial conditions that converge to PO2. The Figure 8 shows an example of this bistability. We simulated two solutions starting at different initial conditions by the MATLAB solver *dde23 *(Shampine and Thompson 2000) for $t=5\times 10^5$. We then graphed the last interval of length t=1000. Comparing the amplitudes of periodic oscillations in this figure to amplitudes in the bifurcation diagram obtained using DDE Biftool in Figure  7, we observed that the solution in the left panel of Figure 8 represents PO1 and in the right panel PO2. This numerical evidence confirms the existence of bistability between periodic orbits.

## Proof of Theorem 1

We prove the Theorem 1 in a series of Lemmas.

### Lemma 2

Consider the ODE system (4) and let $d:=B - \frac{\kappa \gamma _1\gamma _2\gamma _3}{KG\alpha }$. Then

- When d<0 then the equilibrium
  13 $$\begin{aligned} Q:= \left( 0,0, \frac{ B}{\kappa \gamma _3} \right) \end{aligned}$$
  is the only non-negative equilibrium.
- When d>0 then there are two equilibria, *Q* as given in (13) and $P=(R^*,p^*,m_P^*) \in {\mathbb R}^{3+}$.
- When d=0 the equilibria *Q* and *P* coincide.

### Proof

To find equilibria we set $\dot{R}=\dot{p}=\dot{m}=0$ in equations (4) and solve,

14 $$\begin{aligned} \alpha p - \gamma _1 R&= 0 \end{aligned}$$

15 $$\begin{aligned} K GmR -\gamma _2 p&= 0 \end{aligned}$$

16 $$\begin{aligned} \frac{ B}{\kappa + p} - \gamma _3 m&= 0 \end{aligned}$$

For the following calculations, we use two constants

17 $$\begin{aligned} A = \frac{\gamma _2}{K} \quad \text{ and } \quad C = \frac{\alpha }{\gamma _1} \end{aligned}$$

From (15), we solve for the term *GmR* to obtain

$$ GmR = \frac{\gamma _2}{K} p. $$

From (14) we have

$$ R = \frac{\alpha }{\gamma _1} p = Cp. $$

and then we can rewrite *GmR* as

18 $$\begin{aligned} GmR = GmCp = Ap \end{aligned}$$

There are two cases to consider: p=0 and p≠0.

*Case 1:* If p=0 then the equilibrium is defined by

19 $$\begin{aligned} Q:= (R^*, p^*, m_Q^*) = \left( 0,0, \frac{ B}{\kappa \gamma _3} \right) , \end{aligned}$$

where the $m_Q^*$ is the value obtained from (16).

*Case 2:* If p≠0, then from the expression in (18), one arrives at $m_P^* = \frac{A}{GC}$.

With this value fixed, we consider (16) which gives a rational expression for *p* and reads

$$\begin{aligned} \frac{B}{\kappa + p} = \gamma _3 \frac{A}{GC}, \end{aligned}$$

from which

$$\begin{aligned} p^*= \frac{BGC}{\gamma _3 A} - \kappa . \end{aligned}$$

Note that direct computation shows that

20 $$\begin{aligned} p^*>0 \qquad \Longleftrightarrow \qquad d>0, \end{aligned}$$

The equilibrium expression for $R^*$ is given by $R^*=Cp^*$ and thus the unique positive equilibrium is given by

21 $$\begin{aligned} P:= (R^*, p^*, m_P^*) = \left( Cp^*,p^*, \frac{A}{GC} \right) , \end{aligned}$$

Therefore, when d>0, system has two equilibria *Q* and *P*, verifying part (b) of the Lemma. From the same argument as above, it can be noted that $p^*= 0$ when d=0, which completes part (c) of the proof. One can also observe that $d<0 \Longleftrightarrow p^{*} < 0$, which completes the justification for part (a). □

### Lemma 3

Consider the ODE system (4) and suppose that $d=B - \frac{\kappa \gamma _1\gamma _2\gamma _3}{KG\alpha } <0$. Then the equilibrium $Q= \left( 0,0, \frac{B}{\kappa \gamma _3} \right)$ is globally asymptotically stable.

### Proof

Since $\frac{ B}{\kappa + p}< \frac{B}{\kappa }$ we have

$$\begin{aligned} \limsup _{t\rightarrow \infty } m(t) \le \frac{B}{\kappa \gamma _3}. \end{aligned}$$

Hence, without loss of generality we may assume that $m(t) \le \frac{B}{\kappa \gamma _3}$ for all t>0. Since d<0, the matrix

$$M:=\left[ \begin{array}{cc} -\gamma _1 & \alpha \\ KG\frac{B}{\kappa \gamma _3} & -\gamma _2 \end{array}\right] $$

has a negative trace and a positive determinant, and hence *M* is Hurwitz. At the same time, for $\gamma :=\max \{ \gamma _1,\gamma _2\} + 1$, we can write M=X-γI where *X* is a matrix with all positive entries. Therefore principal eigenvalue μ>0 of *X* is real with left positive eigenvector $U=(u_1,u_2) >0$. It follows that the principal eigenvalue λ of *M* has the form $\lambda =\mu -\gamma <0$ and has the same eigenvector *U* satisfying UM=λU. Finally, let $W(t):=u_1 R(t) + u_2 p(t)>0,$ then

$$ \dot{W} = u_1 \dot{R} + u_2 \dot{p} = (-\gamma _1 u_1+ K G m u_2)R+ (\alpha u_1 - \gamma _2 u_2) p$$

$$<(-\gamma _1 u_1+ KG\frac{B}{\kappa \gamma _3} u_2)R+ (\alpha u_1 - \gamma _2 u_2) p=UM (R,p)^T=\lambda W<0.$$

This implies that W(t)→0, and consequently, that R(t),p(t)→0 as $t\rightarrow \infty$. Then it readily follows that $m(t) \rightarrow m^*_Q$ as $t\rightarrow \infty$. □

### Local Stability of Equilibria

Throughout this section, we consider the ODE system (4) and let $d:=B - \frac{\kappa \gamma _1\gamma _2\gamma _3}{KG\alpha }$.

#### Lemma 4

If d<0, *Q* is locally asymptotically stable. If d>0, *Q* is unstable.

#### Proof

Consider the Jacobian of the system (4)

$$ \textbf{J} = \begin{bmatrix} - \gamma _1 & \alpha & 0 \\ K Gm & - \gamma _2 & K GR \\ 0 & -\frac{ B}{(\kappa + p)^2} & - \gamma _3 \end{bmatrix} $$

Then, the Jacobian at $Q:= (R^*, p^*, m_Q^*) = (0,0, \frac{B}{\kappa \gamma _3})$

$$ \textbf{J}_{Q} = \begin{bmatrix} - \gamma _1 & \alpha & 0 & \\ KGm_Q^* & -\gamma _2 & 0 \\ 0 & -\frac{B}{\kappa ^2} & - \gamma _3 & \end{bmatrix} $$

Upon inspection, the third eigenvalue is negative, $\lambda _3 = - \gamma _3$. To compute the other eigenvalues $\lambda _1,\lambda _2$, consider the 2×2 upper submatrix of $\textbf{J}_{Q}$ whose characteristic polynomial is,

$$ \lambda ^2 + (\gamma _1 + \gamma _2)\lambda + \gamma _1 \gamma _2 -KGm_Q^*\alpha =0 . $$

Substituting $m_Q^*=\frac{B}{\kappa \gamma _3}$, we obtain the polynomial $a\lambda ^2 + ab\lambda - d =0$ where

$$\begin{aligned} a=\frac{\kappa \gamma _3}{KG\alpha }>0 , \qquad b=\gamma _1+\gamma _2 >0. \end{aligned}$$

Therefore the remaining two eigenvalues are

22 $$\begin{aligned} \lambda _{1,2} =\frac{-ab \pm \sqrt{a^2b^2+4ad}}{2a} . \end{aligned}$$

We consider the cases d<0 and d>0 separately. When d<0 it follows that $a^2b^2>4ad$, and therefore using (22) either $\lambda _1 <0$ and $\lambda _2 <0$ are real negative eigenvalues or both eigenvalues $\lambda _{1,2} = -\frac{b}{2} \pm i \beta$ for some $\beta \ge 0$ have negative real part. Therefore when d<0, *Q* is locally asymptotically stable since b>0.

When d>0, (22) implies that

$$ \lambda _1 =\frac{-ab - \sqrt{a^2b^2+4ad}}{2a}<0, \quad \lambda _2 =\frac{-ab + \sqrt{a^2b^2+4ad}}{2a}>0 .$$

Thus when d>0 the Jacobian at *Q* has two negative real eigenvalues and one positive real eigenvalue; therefore, *Q* is unstable. □

#### Lemma 5

For all values d>0, *P* is locally asymptotically stable.

#### Proof

Consider the Jacobian at $P:= (R^*, p^*, m_P^*) = \left( Cp^*,p^*, \frac{A}{GC} \right)$

$$ \mathbf {J_P} = \begin{bmatrix} - \gamma _1 & \alpha & 0 & \\ KGm_P^*& -\gamma _2 & KGAp^* \\ 0 & -\frac{B}{(\kappa +p^*)^2} & - \gamma _3 & \end{bmatrix} $$

The characteristic polynomial of $J_P$ is

$$\begin{aligned} \chi _P(\lambda ;d)= \lambda ^3 +\lambda ^2(\gamma _1+\gamma _2+\gamma _3) +\lambda \bigg (\gamma _1\gamma _2+\gamma _1\gamma _3+\gamma _2\gamma _3-\alpha KGm_P^*+\frac{KGAp^*B}{(\kappa +p^*)^2}\bigg )\\ -\alpha KGm_P^*\gamma _3+\gamma _1\gamma _2\gamma _3 + \frac{KGAp^*B \gamma _1}{(\kappa +p^*)^2} \end{aligned}$$

Substituting the expression for $m_P^*=\frac{A}{GC}=\frac{\gamma _1 \gamma _2}{KG\alpha }$ into the equation above and noting that $\alpha KGm_P^* = \gamma _1 \gamma _2$, one can simplify the linear and constant terms in the polynomial to obtain

23 $$\begin{aligned} \chi _P(\lambda ;d) = \lambda ^3 +\lambda ^2(\gamma _1+\gamma _2+\gamma _3) +\lambda \bigg (\gamma _1\gamma _3+\gamma _2\gamma _3 +\frac{KGAp^*B}{(\kappa +p^*)^2}\bigg ) + \frac{KGAp^*B \gamma _1}{(\kappa +p^*)^2}. \end{aligned}$$

For what follows, we have noted the polynomial’s dependence on the parameter *d* explicitly.

The constant term of the polynomial in (23) takes on the same sign as $p^*$, and from equation (20) it follows that $\chi _P(0;d)>0$ for all d>0. Letting

$$c_1:=\gamma _1+\gamma _2+\gamma _3, \quad c_2:=(\gamma _1+\gamma _2)\gamma _3+\frac{KGAp^*B}{(\kappa +p^*)^2}, \quad c_3:=\frac{KGAp^*B\gamma _1}{(\kappa +p^*)^2},$$

we observe that $c_1>\gamma _1>0$ and $c_2> \frac{KGAp^*B}{(\kappa +p^*)^2}>0$.

Therefore,

$$ c_1 c_2> \frac{KGAp^*B\gamma _1}{(\kappa +p^*)^2} = c_3 >0.$$

Using the Routh-Hurwitz criterion, we conclude that all roots of $\chi _P(\cdot ,d)$ have negative real parts for all d>0. Therefore, the equilibrium *P* is locally asymptotically stable for all d>0. □

Having established the local asymptotic stability of the equilibrium *P* in Lemma 5, we are now prepared to proceed with the proof of Theorem 1, which builds upon the findings of the previous Lemmas  2, and  4.

#### Proof of Theorem 1

Proof of the existence of equilibrium solutions *Q* and *P*, as a function of *d*, follows from Lemma 2. The stability of *Q* as a function of *d* follows from Lemma 4 and local asymptotic stability of *P* is a consequence of Lemma 5. The fact that at d=0 the equilibria *P* and *Q* coincide and exchange their stability implies that there is a transcritical bifurcation at d=0. □

## Proof of Theorem 2

In this section we prove the remainder of Theorem 2. We begin by considering the DDE system in (3) with delay τ>0.

### Proof

The equilibria *Q*, *P* for (3) can be computed explicitly as a function of the parameter *d* by solving

24 $$\begin{aligned} \alpha p + (e^{-\mu \tau }-1) GmR - \gamma _1 R&= 0 \end{aligned}$$

25 $$\begin{aligned} K e^{-\mu \tau } GmR -\gamma _2 p&= 0 \end{aligned}$$

26 $$\begin{aligned} \frac{e^{-\mu \tau } B}{\kappa + p} + (e^{-\mu \tau }-1) G mR - \gamma _3 m&= 0 \end{aligned}$$

For the following calculations, we use two constants

27 $$\begin{aligned} A = \frac{\gamma _2}{K e ^{-\mu \tau }} \quad \text{ and } \quad C = \frac{\alpha + (e^{- \mu \tau }- 1)A}{\gamma _1} \end{aligned}$$

From (25), we solve for the term *GmR* to obtain GmR=Ap. Substituting this expression into (24), we have

28 $$\begin{aligned} R = \frac{\alpha p + (e^{- \mu \tau }-1)GmR}{\gamma _1} = \frac{\alpha p + (e^{- \mu \tau }- 1)Ap}{\gamma _1} = Cp, \end{aligned}$$

and then we can rewrite *GmR* as given below.

29 $$\begin{aligned} GmR = GmCp = Ap \end{aligned}$$

Equation (28) shows that the value for *R* is uniquely determined once *p* is computed, and we focus on the calculation of *p* in what follows. There are two cases to consider: p=0 and p≠0.

*Case 1:* If $p^* = 0$, then (28) implies that $R^*=0$, and the equilibrium point is

30 $$\begin{aligned} Q:= (R^*(\tau ), p^*(\tau ), m_Q^*(\tau )) = \left( 0,0, \frac{e^{-\mu \tau } B}{\kappa \gamma _3} \right) , \end{aligned}$$

where the $m_{{\scriptscriptstyle Q}}^*$ is the value obtained from (26).

*Case 2:* If $p^* \ne 0$, then from the expression in (29), one arrives at $m_P^* = \frac{A}{GC}$. Using the definitions of *A* and *C*, the expression for $m_P^*$ can also be written as

31 $$\begin{aligned} m_P^*= \frac{\gamma _1 \gamma _2}{G[(K \alpha +\gamma _2)e^{-\mu \tau }-\gamma _2]}. \end{aligned}$$

Note that $m_P^*> 0$, and therefore biologically relevant, for $\tau \in [0, \tau _{max})$ where

32 $$\begin{aligned} \tau _{\max }:=\frac{1}{\mu } \ln \left( 1+\frac{K \alpha }{\gamma _2}\right) . \end{aligned}$$

Using (25), one can solve for $Gm_P^* R$ in terms of *p* and substitute into equation (26) to reduce to

33 $$\begin{aligned} (e^{\mu \tau }-1) \frac{ \gamma _2}{K} p + \gamma _3 m_P^* = \frac{e^{-\mu \tau } B}{\kappa + p}, \end{aligned}$$

where the left side of (33) is a linearly increasing function of *p* and the right side of the equation is a decreasing function of *p*. For a fixed $\tau \ge 0$, this equation admits a unique positive solution $p^*$ (and thus there exists a unique positive equilibrium *P* for the model in (3)) if and only if the following inequality holds.

34 $$\begin{aligned} 0< m_P^* < \frac{e^{-\mu \tau } B}{\kappa \gamma _3} \end{aligned}$$

The same condition can be equivalently expressed as a single inequality

35 $$\begin{aligned} e^{-\mu \tau } [(K \alpha +\gamma _2)e^{-\mu \tau }-\gamma _2] > \frac{\kappa \gamma _1 \gamma _2 \gamma _3}{GB}. \end{aligned}$$

The left side of the above inequality is a decreasing function of τ which takes on the value of Kα when τ=0 and decreases to zero when $\tau = \tau _{\max }$ from (32). Consequently, if

36 $$\begin{aligned} K \alpha > \frac{\kappa \gamma _1 \gamma _2 \gamma _3}{GB}, \end{aligned}$$

there exists a $\tau _{crit} \in (0, \tau _{\max })$ such that *P* exists only if $0< \tau <\tau _{crit}$. On the other hand, when

$$\begin{aligned} K \alpha \le \frac{\kappa \gamma _1 \gamma _2 \gamma _3}{GB}, \end{aligned}$$

*P* does not exist for any value of τ>0. In both cases, *P* does not exist if τ is sufficiently large. When $p^*$ exists, the expression for $R^*$ is obtained by (28), and the unique positive equilibrium is given by

37 $$\begin{aligned} P:= (R^*(\tau ), p^*(\tau ), m_P^*(\tau )) = \left( Cp^*,p^*, \frac{A}{GC} \right) , \end{aligned}$$

for $\tau <\tau _{crit}$. Since the condition in (36) is equivalent to d>0, we conclude that the system has two equilibria *Q* and *P* in that case. The value of $\tau _{crit}$ can computed explicitly by enforcing equality in equation (35). First define $M:= e^{-\mu \tau }$, and find *M* which satisfies

38 $$\begin{aligned} M^2(K\alpha + \gamma _2) - \gamma _2M - \frac{\kappa \gamma _1 \gamma _2 \gamma _3}{GB}&=0 \end{aligned}$$

39 $$\begin{aligned} U M^2 - M - V&=0 \end{aligned}$$

where

$$\begin{aligned} U := \left( \frac{K\alpha }{\gamma _2} +1 \right) , \qquad V:= \frac{\kappa \gamma _1 \gamma _3}{GB} . \end{aligned}$$

The roots are

$$\begin{aligned} M_{1,2} = \frac{1 \pm \sqrt{ 1 +4UV}}{2U} = \frac{1}{2U} \pm \sqrt{ \left( \frac{1}{2U} \right) ^2 + \frac{V}{U}}. \end{aligned}$$

since U>0,V>0 the positive root is

$$\begin{aligned} M^+ = \frac{1}{2U} + \sqrt{ \left( \frac{1}{2U} \right) ^2 + \frac{V}{U}} \end{aligned}$$

and then $\tau _{crit}$ can be computed as

40 $$\begin{aligned} \tau _{crit} = -\frac{1}{\mu } \ln M^+ . \end{aligned}$$

The value of $\tau _{crit}$ is well defined provided that $M^+ <1$. A straightforward calculation shows that $\frac{1}{2U} + \sqrt{ \left( \frac{1}{2U} \right) ^2 + \frac{V}{U}} <1$ is equivalent to V<U-1, which is equivalent to d>0. This confirms that when d>0, which implies that *P* exists, there is an associated $\tau _{crit}$ at which $P(\tau _{crit})$ is on the boundary of the feasible region.

Continuing with equation (31) and considering $m_P^*$ to be a function of *M*, we observe that $\frac{d }{dM} \big ( m_P^{*} \big )<0$. Similarly, one can use equation (33) to define a function of two variables, *F*(*p*, *M*) equivalently as

$$ F(p,M):= (\frac{1}{M}-1) \frac{ \gamma _2}{K} p + \gamma _3 m_P^*(M) - \frac{M B}{\kappa + p}=0. $$

Since

$$ \frac{\partial F}{\partial M} <0, \quad \frac{\partial F}{\partial p} >0,$$

this equation has a unique solution $p^*=p^*(M)$ such that $\frac{d }{d M} \big (p^*\big )>0$ by the Implicit Function Theorem. Furthermore, $p^*(M)>0$ if and only if F(0,M)<0, or equivalently, if $\gamma _3 m_P^*(M) < \frac{M B}{\kappa }.$ In particular, the positive equilibrium exists as long as $M>M^{+}$ where $\gamma _3 m( M^{+}) = \frac{ M^{+} B}{\kappa }$, which is equivalent to $p(M^{+})=0$. The third component of the equilibrium is given by

$$ R(M) =\frac{ M \gamma _2 p(M)}{K Gm(M)},$$

and it is clear that $R^{\prime }\left(M\right)>0$ and $$R(M^{+})=0.$$ Finally, observe that $$M(\tau ) \downarrow M^{+}$$ when $$\tau \uparrow \tau _{crit}$$ from (40). In other words, as we increase the value of the delay from zero to the threshold value of $$\tau _{crit}$$, the positive equilibrium of the DDE system converges to the limit

$$ \lim _{M \rightarrow M^{+}} (R(M),p(M),m(M)) = (0,0,m_P^*),$$

Note that as $d \rightarrow 0^{+}$, the inequality in (35) can only be satisfied for $\tau \in [0, \tau _{crit})$ with $\tau _{crit} \rightarrow 0^{+}$. That is, the equilibrium *P* exists only for $0< \tau < \tau _{crit}$. Then, in the limit when d=0, the inequality in (35) is no longer satisfied, even with τ=0, and the only solution of equation (33) is $p^*=0$ which corresponds to equilibrium *Q*.

Now we consider the stability of *Q* as a function of τ. Note that the positive equilibrium *P* exists as long as $M>M^{+}$ where $\gamma _3 m(M^{+}) = \frac{M^{+} B}{\kappa }$, which is equivalent to $p(M^{+})=0$.

In the limiting case $M=M^{+}$, the stability of the positive equilibrium is determined by the roots of

41 $$\begin{aligned} \chi (\lambda ,d; \tau _{crit} ):=\det \begin{bmatrix} \lambda +\gamma _1+G\hat{m}(1-L M^{+}) & -\alpha & G\hat{R}(1-\hat{L} M^{+}) & \\ -K G \hat{m} \hat{L} M^{+} & \lambda +\gamma _2 & -K G \hat{R} \hat{L}M^{+} \\ G\hat{m}(1-\hat{L}M^{+}) & M^{+} \hat{H} & \lambda +\gamma _3+ G\hat{R}(1-\hat{L}M^{+}) \end{bmatrix}=0,\end{aligned}$$

where the following definitions hold

$$ \hat{L}= e^{-\lambda \tau _{crit} },\quad \hat{m}=m(M^{+}), \quad \hat{R}=R( M^{+})=0, \quad \hat{H} = \frac{B}{(\kappa + p(M^{+}))^2}=\frac{B}{\kappa ^2}.$$

We denote the characteristic polynomial evaluated at $\tau = \tau _{crit}$ using the notation $\chi (\lambda ,d; \tau _{crit} )$, and it simplifies to

$$ \chi (\lambda ,d; \tau _{crit} )=(\lambda +\gamma _3)\left[ (\lambda +\gamma _1+G\hat{m}(1- \hat{L} M^{+}))(\lambda +\gamma _2)- K \alpha G \hat{m} \hat{L} M^{+}\right] .$$

We have that $\chi (\lambda , d; \tau _{crit} )=0$ if $\lambda =-\gamma _3<0$ or if

$$ (\lambda +\gamma _1+G\hat{m}(1-\hat{L} M^{+}))(\lambda +\gamma _2)= K \alpha G \hat{m} \hat{L} M^{+}, $$

which is equivalent to

$$ e^{-\lambda \tau _{crit} }=\hat{L}=\frac{(\lambda +\gamma _1+G\hat{m})(\lambda +\gamma _2)}{G \hat{m} M^{+} (\lambda +\gamma _2 +K \alpha )}.$$

Using equations (27) and (29), we can derive an expression for the quotient

42 $$\begin{aligned} \frac{p}{R}=\frac{(1- e^{-\mu \tau }) Gm + \gamma _1}{\alpha }=\frac{K e^{-\mu \tau } Gm}{\gamma _2}, \end{aligned}$$

From equation (42) evaluated at $M^+= e^{-\mu \tau _{crit}}$, we get

$$ \frac{(1- M^{+}) G\hat{m} + \gamma _1}{\alpha }=\frac{K M^{+} G \hat{m}}{\gamma _2},$$

which can be expressed

$$ \hat{m} G M^{+}=\frac{(G \hat{m}+\gamma _1) \gamma _2}{\gamma _2 + K \alpha }.$$

Substituting, we obtain

$$ e^{-\lambda \tau _{crit} }=\frac{(\lambda +\gamma _1+G\hat{m})(\lambda +\gamma _2)(K \alpha +\gamma _2)}{(G \hat{m} +\gamma _1) \gamma _2 (\lambda +\gamma _2 +K \alpha )} =\left( 1+\frac{\lambda }{C_1}\right) \frac{1+\frac{\lambda }{\gamma _2}}{1+\frac{\lambda }{C_2}},$$

where

$$C_1=\gamma _1+G\hat{m}>0, \quad 0< \gamma _2 <\gamma _2 +K \alpha =C_2.$$

If $\Re {(\lambda )}>0$, then $|e^{-\lambda \tau _{crit} }|<1$, while $|1+\frac{\lambda }{C_1}|>1$ and $|1+\frac{\lambda }{\gamma _2}|>|1+\frac{\lambda }{C_2}|,$ hence

$$ |e^{-\lambda \tau _{crit} }|<1 < \left| \left( 1+\frac{\lambda }{C_1}\right) \frac{1+\frac{\lambda }{\gamma _2}}{1+\frac{\lambda }{C_2}} \right| .$$

Therefore, the equation $\chi (\lambda ,d; \tau _{crit} )=0$ has no roots with $\Re {(\lambda })>0$ and the equilibrium *Q* is stable. The continuity argument shows that the positive equilibrium *P* must be stable for all values of the delay τ that are sufficiently close to the critical value $\tau _{crit}$. Since the positive equilibrium is also stable for the ODE model corresponding to τ=0, a similar continuity argument can be used to conclude that the positive equilibrium *P* must be stable for all sufficiently small values of the delay τ. □

## Discussion

In this paper we study the role of transcriptional and translational delays on the maintenance of ribosome homeostasis in *E. coli.* We derive a delay-differential equation (DDE) model that tracks abundance of free (i.e. not engaged in translation) ribosomes, ribosomal proteins, and ribosomal protein mRNA. This DDE model is obtained by simplifying a 6 dimensional ODE model studied in Shea et al. (2023) and then introducing both transcriptional and translational delays. As in the larger model, the smaller model has two operating regimes. The first regime is the case where there is a single equilibrium *Q* on the boundary of the positive orthant, which corresponds to the biological situation where there are no free ribosomes. The second regime is characterized by the case where the equilibrium *Q* is unstable, and there is an interior equilibrium *P* that is always stable in the corresponding system of 3 ODEs, where delays are set to zero. We investigate whether the delay can destabilize the equilibrium *P*. This task is challenging because the equilibrium *P* only exists for a bounded range of delays $[0,\tau _{crit}]$, where $\tau _{crit}$ depends on several model parameters.

We determine the values of all but 5 parameters from the literature, and we find reasonable bounds on these 5 parameters. The resulting five dimensional product space, along $[0,\tau _{crit}]$ is sampled numerically, and we find that *P* remains stable throughout this space. When the estimated value of the protein decay rate is increased (by one hundred fold) and the resulting parameter space is sampled, we find a small percentage of the parameters where *P* is destabilized by a Hopf bifurcation. For these parameters choices, *P* enters the positive orthant at τ=0 and exits the positive orthant at $\tau _{crit}$ via transcritical bifurcation with the boundary equilibrium *Q*, as a stable equilibrium. Therefore it is not surprising that when *P* undergoes a Hopf bifurcation and loses stability at $\tau _1$ there is a second Hopf bifurcation at $\tau _2 >\tau _1$ where *P* regains its stability. Since for this set of parameters we did not find any secondary Hopf bifurcations, we investigate the question of whether or not the model is capable of supporting such secondary Hopf bifurcations and/or more complex structures of periodic orbits.

After a detailed analysis of the linearization of the system at *P*, we were able to describe a set of Hopf bifurcation curves that suggest that as the growth rate $\mu \rightarrow 0$, the number of Hopf bifurcations increases without bounds. Again, these bifurcations have to come in pairs of delay values $\tau ^i_1<\tau ^i_2$, but the intervals $[\tau ^i_1,\tau ^i_2]$ are neither nested, nor disjoint. We have shown that the overlap of these intervals may lead to bistability between two stable periodic orbits. Furthermore, the structure of the bifurcations suggests that there may be a stable torus of solutions bifurcating from one of the two stable periodic orbits. Investigating and analyzing full richness of the structure of periodic orbits is beyond the scope of this paper.

Returning to the original biological question of whether the delays can explain the experimental data reproduced in Figure 1, which seem to suggest decaying oscillations, we investigate the number of parameters in the biologically relevant five dimensional parameter space that support decaying oscillations towards *P*. Remarkably, while there are no parameters that would support sustained oscillations, over 50% of the parameters sampled show mildly damped oscillations with very slow decay rate determined by the growth rate and the ribosomal protein decay rate. We conclude that the experimental data in *E. coli* is consistent with the delay-induced slowly decaying oscillations that the analysis of the proposed model suggests are ubiquitous in the parameter space. However, the observed decay rate of the amplitude of oscillations is likely caused by de-synchronization of the experimental cell population. From the mathematical point of view, we find a rich structure of paired Hopf bifurcations as $\mu \rightarrow 0$, which supports bistability between periodic orbits and a potential for stable torus of recurrent solutions.

## Data Availability

Simulation code available upon request.

## Parameter values justification

In order to parameterize the model we use numbers for *E. coli*. Since this is a simplification of the model (1) discussed in Shea et al. (2023), we can make use of some of the derivations of parameters made for (1). There are two challenges to successful parameterization. The first is that the numbers depend on growth condition of *E. coli* so that some of the parameters (i.e. number of ribosomal RNA in a cell) may change by two orders of magnitude. The second challenge is that our model, as every model, is a simplification and thus many parameters have to be interpreted appropriately in terms of measured data. Below is the table of parameter ranges followed by detailed justification of these values.

- First, we describe how to convert number per cell to units of micromolar μM. As an example we take the number of copies of rrn gene in *E. coli*, which is 7 (Condon et al. 1995). Since the volume of *E. coli* is about 1 fL (Moran et al. 2010)(BNID 101788), which is $10^{-15}L$ there are $7 \times 10^{15}$ rrn genes per liter. Dividing by the Avogadro number $6.02214076*10^{23} mol^{-1}$ we get concentration $1.1624 * 10^{-8} mol/L$. Converting the units to micromolar, which is equivalent by multiplying by $10^6$ we get 0.011624μM=11.624nM. This conversion is close to “rule of thumb" conversion (BNID 102068) which says that 1 molecule in *E. coli* corresponds to concentration of 1*nM*,  which would have given concentration of 7*nM*.
- We have $\alpha = \hat{\alpha }r$ where *r* is average number of ribosomal RNA in a cell and $\hat{\alpha }$ was estimates in Shea et al. (2023) as $\hat{\alpha } \in [0.007,0.754]$. We now estimate the range of rRNA concentration in cell.
- There are 2400-7800 copies of mRNA per *E. coli * cell, depending on the growth medium  (Moran et al. 2010) (BNID 112795). This corresponds to concentration range 3.98-12.95μM . The paper (Gausing 1977) measured the fraction of the total transcript which is ribosomal RNA by hybridization of pulse-labeled total cellular RNA to DNA from a transducing λ-phage carrying a complete set of rRNA genes. The relative ratio of rRNA transcript increases from 0.27-0.67 over the range of growth rates studied. This is about three times than 9-22% estimated above and gives 0.27∗3.98=1.0746 and 0.67∗12.95=8.6765 all in μM. The range of *r* is [1.0746, 8.6765]. This gives α in the range [0.0075222, 6.542081]
- (Shea et al. 2023)$\beta = 0.14\; min^{-1} = 0.00233 \; s^{-1}$ Degradation rate of mRNA and rRNA. In *E. coli* mRNA lifespan is about 5 minutes (Milo and Phillips 2016)(BNID 106869).
- (Shea et al. 2023) $\xi =0.014 \; min^{-1} = 0.000233 \; s^{-1}$ Degradation rate of proteins. Rapidly degraded proteins in *E. coli* have lifespan about an hour or 10 times that of mRNA will do 50 minutes  (Milo and Phillips 2016)(BNID 108404).
- (Shea et al. 2023) *K* is not constant since it depends on the nutrient level *I*. Its range has been estimated in from (Shea et al. 2023).
- Our constant *B* is related to parameters in Shea et al. (2023) by
  $$\begin{aligned} B=\frac{B_{max}n}{\kappa _3 + n}\frac{1}{\kappa _4 + g}, \end{aligned}$$
  where *n* is average concentration of NTP in a cell and *g* is average concentration of ppGpp. The parameters in Shea et al. (2023) have the following values
  1. $$B_{max} \in [30, 339]$$.
  2. We derived $I_0 = 6212 \mu$ M as a concentration of NTP required for balanced growth. We use this number as value for *n*.
  3. We selected half-saturation constants of the same order of magnitude $\kappa _3 \in [10^3,10^4] \mu M$
  4. During the transition from exponential to stationary phase the ppGpp concentration is between 30-113μ M (Buckstein et al. 2008) . This is *g*.
  5. Finally, we selected matching half-saturation constant $\kappa _4 \in [30,113] \mu M$
  Therefore $$B\in [ 0.43067110371,0.57475943745 ] s^{-1}$$ .
- *G* is rate of ribosomal protein production. It has been estimated in Shea et al. (2023) as a product $G= \omega \hat{a}$ where ω is the rate of formation of complex *mR* to produce a a representative ribosomal protein and $\hat{a}$ is the $1/K_D$ where $K_D$ is the dissociation constant for binding ribosomes to ribosomal mRNA in units $(\mu M)^{-1}$. Each of these was estimated independently and then ranges were multiplied.
- The growth rate μ of *E. coli* depends on resources, temperature and the strain. We use here the rate $$0.65 h^{-1} $$ reported for *E. coli* MG1655 in  Carlson et al. (2018).

**Table 1:** Parameter values for the model (3)

Parameter	Units	Value/Range	Interpretation
β	$s^{-1}$	0.00233	Degradation rate of mRNA
ξ	$s^{-1}$	0.000233	Degradation rate of proteins.
μ	$s^{-1}$	0.00018	Maximal growth rate of *E. coli* MG1655
α	$s^{-1}$	[0.0075222, 6.542081]	Rate of ribosome assembly
K=K(I)	Unitless	[0, 10.295]	*K* is not constant since it depends on nutrient level *I*
*B*	$$s^{-1} $$	[0.43, 0.57]	mRNA transcription initiation rate
κ	Unitless	[11.29, 119.56]	Michealis-Menten constant for protein repression
*G*	$s^{-1} (\mu M)^{-1}$	[0.7689, 48.8928]	Rate of ribosomal protein production.
